# Extracting Caprolactam from PA6 Waste: Progress in Chemical Recycling and Sustainable Practices

**DOI:** 10.3390/polym18080940

**Published:** 2026-04-11

**Authors:** Damayanti Damayanti, Mega Pristiani, Ho-Shing Wu

**Affiliations:** 1 Department of Chemical Engineering and Materials Science, Yuan Ze University, 135 Yuan-Tung Road, Chung-Li, Taoyuan 320315, Taiwan; damayanti@tk.itera.ac.id (D.D.); megapristiani23@gmail.com (M.P.); 2 Department of Chemical Engineering, Institut Teknologi Sumatera, Jl. Terusan Ryacudu, Way Huwi, Kec. Jati Agung, Lampung Selatan 35365, Lampung, Indonesia

**Keywords:** polycaprolactam, nylon, caprolactam, chemical recycling, mechanical recycling, sustainability, polyamide 6

## Abstract

This review critically evaluates current PA6 recycling technologies, with a specific focus on caprolactam-oriented chemical recycling pathways, including hydrolysis, pyrolysis, glycolysis, ammonolysis, hydrothermal treatment, ionic-liquid-assisted depolymerization, and microwave-assisted processes. Reported caprolactam yields vary significantly depending on reaction conditions and catalyst systems, ranging from below 60 wt% in conventional hydrolysis to above 90 wt% under optimized catalytic, hydrothermal, or microwave-assisted conditions. Among these approaches, microwave-assisted hydrolysis and catalytic depolymerization have emerged as particularly promising, offering substantially reduced reaction times (minutes rather than hours), improved energy efficiency, and high monomer selectivity at moderate temperatures (typically 200–350 °C). This review integrates kinetic modeling approaches, analytical methods for monitoring depolymerization, and downstream separation considerations that govern monomer purity and recyclability. Key challenges, including energy demand, feedstock contamination, scalability, and economic competitiveness, are critically discussed in relation to industrial implementation. Overall, hydrolysis-based and microwave-assisted chemical recycling routes are the most viable pathways for closed-loop recycling of PA6. Future progress will rely on integrated reaction–separation–repolymerization designs, catalyst optimization, and process intensification to enable sustainable and industrially relevant PA6 circularity.

## 1. Introduction

Polyamide 6 (PA6) is a versatile engineering thermoplastic widely used in automotive, construction, separation processes, textiles, and other industries for its exceptional mechanical and thermal properties, strength, and durability. It is a widely used plastic, with a global market exceeding 8 million tons annually and projected to grow at 2.2 wt% per year, reaching 10.4 million tons, valued at approximately 47 billion USD [1,2]. Meanwhile, in the production of PA6, 10 mol% of the reaction mass remains as monomer caprolactam (CL) or as CL oligomers, because the equilibrium of CL polymerization is 90 mol%. That means, during production, this process generates waste PA6 and CL. This non-polymerized fraction must be removed from the polymers, as it would interfere with subsequent processing into end products such as films or engineering plastics [3,4].

From a sustainability perspective, PA6 presents a particularly compelling case for circular material management. Unlike many commodity polyolefins, PA6 is synthesized from CL via equilibrium-limited polymerization, in which approximately 10 mol% of the monomer remains unreacted and must be removed during production [5,6]. The industrial production of PA6 proceeds via hydrolytic ring-opening polymerization of CL, which is inherently a non-equilibrium process involving reversible reactions among monomer, oligomers, and polymer chains [7]. In addition, significant quantities of post-industrial and post-consumer PA6 waste are generated from fibers, carpets, fishing nets, and engineering plastics. These streams contain chemically recoverable amide linkages, making PA6 intrinsically suitable for depolymerization-based recycling routes aimed at monomer recovery rather than down-cycling.

Conventional mechanical recycling remains the dominant industrial practice for PA6 waste management due to its relative simplicity and low capital cost. However, repeated thermal and mechanical processing leads to molecular weight degradation, property deterioration, and limited recyclability, particularly for contaminated or multi-component waste streams. As a result, mechanically recycled PA6 is often relegated to lower-value applications, undermining long-term circularity [8]. In contrast, chemical recycling strategies seek to depolymerize PA6 into its original monomers or well-defined intermediates, enabling the regeneration of virgin-quality materials and offering a fundamentally closed-loop solution.

**Figure 1:**
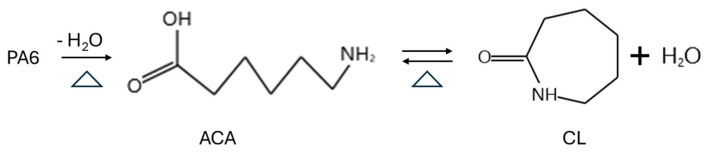
Reaction pathway for PA6 depolymerization.

Over the past two decades, a wide range of chemical recycling pathways for PA6 have been explored, including hydrolysis, pyrolysis, glycolysis, ammonolysis, hydrothermal treatment, ionic-liquid-assisted depolymerization, and, more recently, microwave-assisted processes [9,10]. These approaches differ substantially in reaction severity, energy demand, catalyst requirements, product selectivity, and scalability. Among them, hydrolysis-based routes, particularly those targeting CL or its linear precursor 6-aminocaproic acid (ACA), have demonstrated the highest industrial relevance due to their compatibility with existing polymerization infrastructure. Meanwhile, emerging technologies such as microwave heating and catalytic depolymerization under milder conditions have shown promise in enhancing reaction efficiency, reducing energy consumption, and improving monomer yield and purity. The reaction mechanism of PA6 depolymerization is shown in Figure 1.

Despite the growing body of literature on PA6 recycling [1,11,12], existing reviews often address polymer recycling in a broad, general manner or focus on individual techniques without integrating reaction mechanisms, kinetic behavior, separation strategies, and closed-loop feasibility. A systematic and critical comparison of CL-oriented chemical recycling routes, particularly those emphasizing process intensification, kinetic control, and sustainable energy input, remains limited. Moreover, the connection between depolymerization chemistry and downstream monomer recovery, purification, and repolymerization is frequently under-represented, even though these steps ultimately determine industrial viability.

In this context, this review provides a comprehensive and critical overview of PA6 recycling technologies, with a specific focus on chemical depolymerization pathways for caprolactam recovery. Particular emphasis is placed on hydrolysis-based processes, microwave-assisted techniques, and hydrothermal reactions, along with their associated reaction kinetics and energy characteristics [13]. Mechanical recycling is discussed for comparison, highlighting its advantages and intrinsic limitations. In addition, analytical methodologies for monitoring depolymerization, recent advances in catalyst and reactor design, and strategies for closed-loop PA6–CL recycling are systematically examined [14].

By integrating reaction chemistry, kinetic modeling, process considerations, and sustainability perspectives, this review aims to clarify the current state of PA6 chemical recycling, identify key technical bottlenecks, and outline future research directions required to transition from laboratory-scale demonstrations to industrially viable circular recycling systems.

## 2. Closed-Loop Opportunity for PA6

PA6 holds a significant commercial presence, accounting for approximately 4 wt% of the global consumption of major polymers. PA6 is valued for its favorable rheological properties and low melting temperature. Approximately 54 wt% of the polyamide produced is PA6, while polyamide-6,6 accounts for around 36 wt% [15]. Nearly 75 wt% of PA6 is used in fiber applications, while around 15 wt% is utilized as an engineering plastic. Typical fiber applications include carpets, apparel, home furnishings, and industrial uses [16]. Engineering applications of PA6 range from wheel covers, handles, radiator end tanks, and fuel hoses in the automotive industry to hairdryers, lawnmowers, gears, and bearings. PA6 fibers are commonly used in textiles, fishing lines, and carpets. At the same time, PA6 films are used in food packaging for their toughness, low gas permeability, and temperature resistance, making them suitable for boil-in-the-bag packaging. Additionally, PA6 molding and extrusion compounds are widely used as metal replacements in automotive components.

Nylon intake manifolds are corrosion-resistant, lighter than aluminum (once tooling costs are covered), and offer improved airflow due to their smooth internal bore, even compared to roughcast aluminum. Its self-lubricating properties make it ideal for gears and bearings. Nylon’s electrical insulation, corrosion resistance, and toughness also make it suitable for high-load electrical components such as insulators, switches, and cable ties. Additionally, nylon is commonly used for power tool housings due to its durability and strength [17].

Primary recycling, or depolymerization, breaks down polymer waste into its original monomers, producing materials of equivalent quality to virgin polymers. However, stringent fuel efficiency and CO_2_ regulations are driving shifts toward alternative materials. PA6 is used in films and coatings to prevent corrosion. At the same time, PA6 is increasingly adopted for its durability, lightweight, versatility, and design flexibility, enabling the creation of advanced vehicle forms without compromising safety or stability. Nylon’s environmental benefits align with global emission regulations, encouraging its broader use in automobiles.

## 3. Environmental Problems of PA6 Waste

PA6-based waste poses some environmental problems, such as microplastic pollution in aquatic environments (from fishing nets and synthetic textile fibers from laundering) [18]. The ecological impact of PA6 waste can be significant due to the following issues:

(i) Microplastic pollution: Synthetic fibers such as PA6 degrade into microplastics, which can contaminate water bodies. These fragments are ingested by aquatic organisms, threatening marine life and disrupting ecosystems [19].
(ii) Non-biodegradability: PA6 does not break down naturally, remaining in landfills and the environment for decades and causing lasting ecological harm. This complicates its disposal [20].
(iii) Cost constraints: The purification of recovered monomers is a critical economic factor. High-purity caprolactam is required for repolymerization into “virgin-quality” PA6, often involving vacuum distillation or solvent-based recovery methods [21].
(iv) Feedstock contamination: Recycling streams often contain multiple types of plastics or contaminants like dyes, adhesives, and other additives that complicate the process. Chen et al. presented a green, simple, and cost-effective method for the closed-loop chemical recycling of PA6 to its monomer CL and back to PA6, achieving high yield (88 mol%) and purity (97 mol%). Kinetic and theoretical mechanistic investigations revealed that this alkali-catalyzed depolymerization proceeds via deprotonation of amide groups, followed by intramolecular cyclization to form lactam units, which sequentially drop from the chain end [22].
(v) High energy consumption and greenhouse gas emissions: PA6 production is energy-intensive and depends on fossil fuels. Polymerization of CL emits greenhouse gases that contribute to global warming. Because PA6 can be converted back into its base monomer with high selectivity, chemical recycling is often preferred over general pyrolysis, which tends to produce lower-value decomposition products [21].
(vi) Challenges in recycling: Recycling PA6 is challenging due to its complex structure and contamination in post-consumer products (e.g., textiles blended with other fibers). While mechanical and chemical recycling processes exist, the overall recycling rate remains low, and much of the PA6 waste ends up in landfills. The quality of the recycled material may be lower than that of the original polymer, so it is often used for less demanding applications (such as plastic). This process can be performed with post-industrial materials, such as scrap or processing waste, and post-consumer nylon waste [23].
(vii) Landfill overload: PA6 waste contributes to landfill accumulation, mainly when disposed of as part of non-recycled consumer products like carpets, apparel, and packaging materials. Landfills cannot break down these synthetic polymers, leading to long-term environmental contamination [24].
(viii) Water and soil contamination: PA6 waste that finds its way into the environment can leach chemicals into water sources or soil, posing risks to aquatic ecosystems and soil health. Over time, these contaminants can affect wildlife and human populations throughout the food chain [25].

## 4. The Common Recycling of PA6

**Figure 2:**
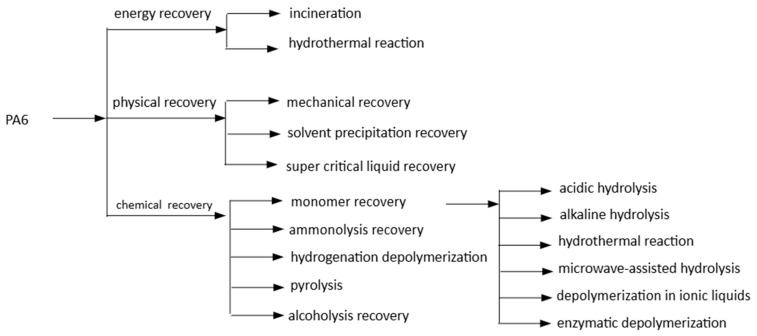
PA6 recycling process scheme.

The chemical structure of a polymer plays a crucial role in determining its end-of-life pathways [26]. Polymers with carbon–carbon backbones, such as polyethylene and other polyolefins, require extremely high temperatures for depolymerization and show low monomer selectivity due to random chain scission. However, the strength of these bonds makes them highly suitable for mechanical recycling. In contrast, polymers with backbones containing C–X bonds (where X is a heteroatom) are more susceptible to selective cleavage, allowing depolymerization under milder conditions. Consequently, depolymerization research mainly focuses on polyesters [27], polyamides, polyurethanes, and polycarbonates [12]. PA6-based products recycled included the recycling streams of carpets [28,29], fishing nets [30,31], and post-industrial PA6 fiber waste, but not typical recycled products: textiles and apparel (e.g., activewear, tights, windbreakers), packaging, electronics, and industrial components(gears, bushings, and rollers used in machinery). Establishing efficient, long-term recycling solutions for plastics such as PA6 is crucial. Currently, most plastic recycling relies on mechanical recycling, where plastic waste undergoes mechanical processing, such as melting, to be transformed into new products, like converting plastic bottles into fibers. However, mechanical recycling often leads to degraded plastic quality and limits the number of recycling cycles. In contrast, chemical recycling involves depolymerizing plastic into its original monomers, producing virgin-quality plastic without loss of quality [32,33]. Pyrolysis and aminolysis are PA6’s most commonly documented chemical recycling techniques; all involve harsh reaction conditions (temperatures between 250 and 300 °C) and do not provide effective PA6 regeneration from damaged polymers [34,35]. A possible solution is the dissolution/precipitation approach [36]. As shown in Figure 2, PA6 recovery can be divided into energy, physical, and chemical recovery [31].

For this reason, chemical recycling is the only way to close the loop in plastic manufacturing and is a sustainable recycling model. In addition, chemical recycling can reduce marine and land pollution and incorporate the circular economy principle [9,11]. Hydrolysis is a type of chemical recycling that converts waste into new building blocks, such as monomers, oligomers, and functional chemicals, to produce new polymers. The material’s value can be maintained or even increased in some cases.

Several previous investigations have examined the hydrolysis of PA6 in different solvents to obtain CL [14,37]. They investigated the effects of reaction time and solvent type (including hydrochloric acid, phosphoric acid, and formic acid) on the liquefaction and acidic degradation of PA6 [13]. They found that acid hydrolysis produces monomers such as ACA. The degradation of PA6 using phosphoric acid (H_3_PO_4_) in different concentrations and reaction times in the microwave reactor. In different media, the yield of CL was more than 55 wt% of the PA6 under stirring at 400 °C [38]. In recent years, microwave heating has emerged as a promising technique for polymer degradation due to its volumetric and selective heating capabilities. This method enables rapid heating in cold environments, thereby preserving product quality by minimizing secondary reactions and reducing energy consumption [39].

Table 1 outlines the advantages and disadvantages of various PA6 recycling methods. PA6 is prone to hydrolytic degradation, with non-enzymatic random chain scission as a primary driver. This degradation typically occurs through acid- and base-catalyzed ester hydrolysis, leading to PA6 chain breakdown. Ye et al. reported a highly efficient metallocene catalytic system based on earth-abundant early transition and lanthanide metals, achieving PA6 depolymerization rates up to 810 CL mol(Cat.)^−1^·h^−1^ at 240 °C with ≥99 mol% yield. This solventless process operates with catalyst loadings as low as 0.04 mol% and temperatures as low as 220 °C, marking the mild PA6 depolymerization conditions [40]. A degradation review of PA6 to monomers using different catalysts reported in previous work is presented in Table 2.

Several previous investigations have examined the hydrolysis of PA6 using different solvents to obtain CL [51,52]. They investigated the effects of reaction time and type of solvents, such as hydrochloric acid, phosphoric acid, and formic acid, on the liquefaction acidic degradation of PA6 [13]. They found that acid hydrolysis produces monomers such as ACA. The degradation of PA6 using phosphoric acid in different concentrations and reaction times in the microwave reactor. Across different media, the CL yield exceeded 55 wt% of the PA6 under stirring at 400 °C [38]. Microwave heating has recently emerged as a promising technique for polymer degradation, owing to its volumetric and selective heating properties, enabling rapid heating even in cold environments. This approach helps preserve product quality by minimizing secondary reactions and can also reduce energy consumption [39].

Determination of PA6, CL, and ACA during the reaction is crucial for PA6 recycling. Kembłowski and Torzecki presented a two-point rheometrical method for determining the weight-average molecular weight (Mw) of PA6 [53]. Shukla et al. determined the PA6 molecular weight using relative viscosity with an Ostwald U-tube viscometer [48,54,55]. NH2 end-group analysis was performed to measure the average molecular weight of PA6 in accordance with ASTM D 2074-07 [50,56]. The products obtained from PA6 recycling were analyzed using nuclear magnetic resonance spectroscopy [13]. Kulkarni and Kanekar used high-performance liquid chromatography to analyze the CL waste from PA6 production plants. The main components were CL, ACA, and their linear and cyclic oligomers [57]. Žagar et al. presented the quantitative determination of PA6 and PA66 Content in polyamide-containing wastes [58].

Table 3 offers a comparative overview of key PA6 recycling technologies focusing on selectivity, energy use, and product purity. This table allows for a side-by-side assessment of engineering practicality and sustainability, aiding in the identification of near-term industrial opportunities while highlighting techniques still mainly at the academic research stage. Hydrolysis-based methods are the most developed for industrial applications because they yield high monomer recovery and are compatible with existing polymerization systems, despite needing significant energy for downstream purification. Microwave-assisted and catalytic depolymerization methods show great potential for process intensification by reducing reaction times and operating temperatures. On the other hand, pyrolysis provides feedstock flexibility but has lower selectivity and presents more complex separation challenges. Glycolysis and ammonolysis are limited by incomplete monomer recovery and by-product formation, while ionic-liquid and enzymatic methods are constrained by cost, scalability, or reaction speed. Overall, the practicality of these processes primarily depends on separation efficiency and system integration, rather than depolymerization yield alone.

To contextualize laboratory-scale advances, representative industrial implementations of PA6 recycling are summarized in Table 4. Current industrial practice is dominated by hydrolysis-based depolymerization routes, exemplified by Aquafil’s ECONYL^®^ process, which enables closed-loop regeneration of CL from post-consumer waste streams such as carpets and fishing nets. Several chemical companies, including DOMO Chemicals and BASF, are developing integrated recycling platforms that combine depolymerization, purification, and repolymerization within existing polymer production infrastructure. While pyrolysis-based approaches offer flexibility for mixed plastic waste, they generally lack monomer selectivity compared to hydrolysis routes. Emerging strategies, such as hybrid mechanical–chemical recycling and bio-integrated processes, are also under development. Overall, industrial deployment remains limited, but it shows that hydrolysis-based chemical recycling is currently the most technologically advanced pathway for producing circular PA6.

### 4.1. Mechanical Recycling

A comprehensive review of current polymer recycling pathways, including mechanical and chemical methods, covers industrial technologies, design strategies, and examples of recycling specific waste streams [33].

**Figure 3:**
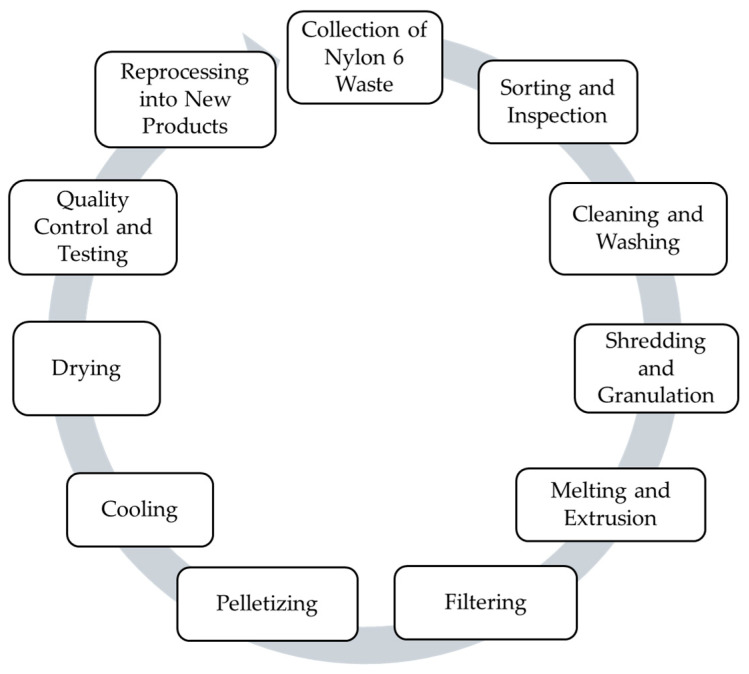
Mechanical recycling process of PA6.

Mechanical recycling is a physical process in which plastic waste is converted into flakes, granules, or pellets of sufficient quality for manufacturing. These recycled materials are melted and reshaped into new products through extrusion [64]. Mechanical recycling preserves the molecular structure of plastics by mechanically crushing and remelting them into granules suitable for manufacturing new products. It is most effective when the plastic waste consists of a single polymer type, making it a preferred method for recycling in such cases [65]. Mechanical recycling is often costly, and the recycled plastic end product is typically more expensive than virgin plastic. The process generally involves removing contaminants, breaking the waste into smaller pieces, grinding or converting it into flakes or pellets, and then washing, drying, and melting for reuse [66]. The effects of reprocessing cycles are examined, with particular attention to the impact of moisture on polyamides before and after recycling [67]. The mechanical recycling process is shown in Figure 3.

Liu and Bertilsson demonstrated the conditions for mechanical recycling: The material was dried and then extruded using a corotating twin-screw extruder at 240 °C and 200 rpm. After extrusion, it was injection molded at 240 °C with a mold temperature of 50 °C [68]. Abdelwahab et al. demonstrated a strategy for reprocessing post-consumer recycled carpet waste containing polyamide, polypropylene, and other additives and reinforcing agents by blending it with PA6 at various weight ratios via reactive extrusion [29,69]. The benefits of mechanical recycling of PA6 are as follows:

(i) Environmental impact: Reduces landfill waste and conserves resources by reusing existing materials.
(ii) Energy Efficiency: Generally, it consumes less energy compared to producing virgin PA6 from raw materials.
(iii) Economic advantages: Can be cost-effective due to lower material costs and potential regulatory incentives.
(iv) Quality degradation: Mechanical recycling may cause some loss in mechanical properties due to thermal degradation.
(v) Contamination: Other plastics or contaminants can affect the quality of recycled PA6.
(vi) Market Demand: The success of recycling efforts depends on the demand for products made from recycled materials.

### 4.2. Chemical Recycling

**Figure 4:**
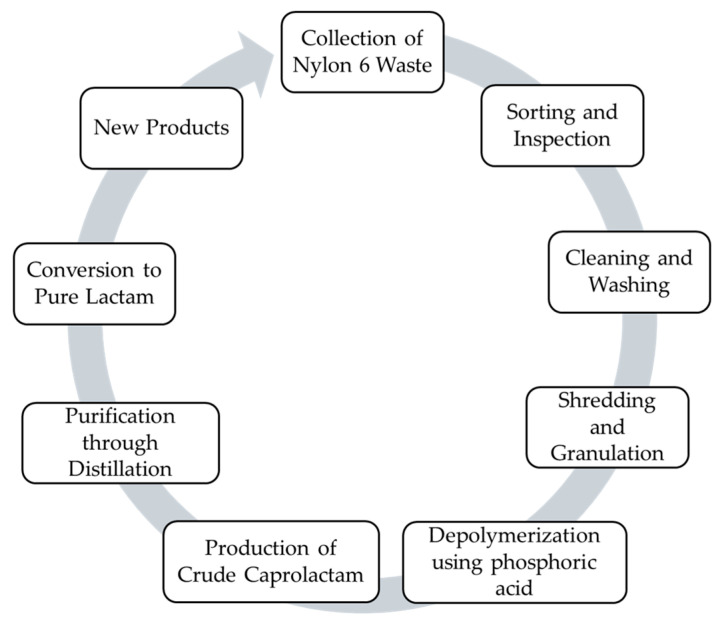
Chemical recycling process of PA6.

Figure 4 shows the chemical recycling process of PA6. Chemical recycling has gained increasing attention as a strategy to reduce environmental pollution and support the transition to a circular economy. It can complement mechanical recycling by breaking down polymers into their monomeric forms, providing an alternative for waste streams unsuitable for mechanical processing [9]. Polymers are chemically converted into monomers through specific reactions, allowing these monomers to be used to reproduce the original polymer or to create a related polymeric product [70]. Chemical recycling remains a challenging process requiring significant investment and specialized expertise. As a result, it is not yet fully developed, with only a limited number of companies currently advancing it [71]. Some chemical reactions that decompose polymers into monomers are oxidation, glycolysis, pyrolysis, ammonolysis, hydrogenation, hydrolysis, gasification, cracking, and methanolysis [72].

Although detailed kinetic data for PA6 depolymerization are limited and often system-specific, reaction kinetics are crucial for determining process performance, particularly with respect to reaction time, temperature dependence, and reactor design. In hydrolysis-based systems, the rate of amide bond cleavage is strongly influenced by temperature, water concentration, and catalytic conditions, whereas in pyrolysis and catalytic depolymerization, kinetics are governed by radical or chain-end mechanisms.

#### 4.2.1. Pyrolysis

Pyrolysis is the thermal decomposition of a substance through heating alone. CL was the only reported product in the non-catalyzed pyrolysis of PA6. The reaction occurred at temperatures between 350 °C and 500 °C, but the product yield and purity were not documented [73]. It was observed that the formation of CL occurs more rapidly when generated at the chain end than in the middle of the polymer chain. CL remained the primary product during high-temperature pyrolysis of PA6 at 800 °C [74,75]. With the assistance of bases, pyrolysis has emerged as a promising method for depolymerizing PA6. Mukherjee et al. demonstrated this by using solid sodium hydroxide (NaOH) in a batch reactor for 4.5 h, achieving a 90.5 wt% yield of CL from PA6 [76]. A vacuum process was used to remove the CL and enhance the forward reaction rate of the equilibrium reaction. Czernik et al. reported that catalytic pyrolysis is an efficient method for recovering CL from PA6, achieving yields of 85 wt% or higher at 330–360 °C using potassium hydroxide on α-alumina as the catalyst [77].

Bryson studied the recovery of monomers from nylon carpets via catalytic depolymerization/pyrolysis in a reactive extruder [78]. Extrusion of a 100:1 ratio of pure PA6 and KOH was done in a 30 mm counterrotating non-intermeshing twin screw extruder. Yang et al. reported catalytic pyrolysis of PA6 using γ-Al_2_O_3_-supported metal catalysts (Ni, Cu, Fe, or Co). The γ-Al_2_O_3_ enhanced the CL yield to 69.0 wt% through base-catalyzed PA6 depolymerization and intramolecular cyclization. In the presence of metal catalysts, the CL yield further increased to 73.3 wt% [79].

(A) Thermal degradation kinetics using thermal gravimetric analysis

Thermogravimetric analysis (TGA) has proven effective for examining the thermal stability of polymeric systems. Studying thermal degradation and decomposition behavior under heat is highly recommended for optimizing kinetic parameters [80]. TGA is a high-temperature furnace technique used to measure a sample’s mass loss over time as the temperature is varied, making it widely used in kinetic studies. TGA uses a minimal sample weight to minimize mass and heat-transfer barriers, ensuring more accurate and reliable reaction-rate data than other methods [81]. Another method may vary depending on the composition and concentration of the polymers’ main components.

TGA is commonly used to analyze the thermal behavior of materials during pyrolysis, providing insights into their decomposition processes. It is particularly effective for determining the kinetics of polymer thermal degradation, as it tracks mass loss over time and temperature during the reaction for PA6 decomposition:

(1) $${\mathrm{PA}6}_{\left(\mathrm{solid}\right)}\;\overset{k}{\to }\mathrm{Volatile}\;{\mathrm{matters}}_{\left(\mathrm{gas}\right)}\;+\;{\mathrm{Char}}_{\left(\mathrm{solid}\right)}$$

where volatile matters mean the total of the gas, tar, and k is the rate constant of the reaction, which depends on temperature and is expressed by the Arrhenius equation:

(2) $$k=Ae^{\left(-E_{a}/RT\right)}$$

where E_a_ is the activation energy (kJ/mol), T is the temperature (K), R is the gas constant (8.314 J/K mol), and A is the preexponential factor (min^−1^). The rate of decomposition is shown in Equation (3).

(3) $$\frac{d\alpha }{dt}=k\left(T\right)f\left(\alpha \right)$$

where t, k(T), and f(α) represent the time, the rate constant, and the reaction model, respectively, and α is the conversion based on the weight loss of decomposed biomass and is defined as

(4) $$\alpha =\frac{m_{i}-m_{a}}{m_{i}-m_{f}}$$

where m_i_ is the initial mass, m_a_ is the actual mass, and m_f_ is the mass after pyrolysis of the sample. By combining Equations (3) and (4), we can get the fundamental analytical methods of kinetic analysis based on TGA data, expressed by

(5) $$\frac{d\alpha }{dt}=Aexp\left(-\frac{E_{a}}{RT}\right)f\left(\alpha \right)$$

where A and Ea are the frequency factor and activation energy, respectively.

Herrera et al. reported that the thermal degradation of polyamides occurs in one step in nitrogen and in two steps in air, with major products including CO_2_, H_2_O, NH_3_, and toxic HCN. The kinetic parameters of the degradation process were analyzed using dynamic thermogravimetric measurements. The apparent activation energy for the first-order reaction was 162 kJ/mol [82].

For the single-reaction model with n reaction orders, the reaction rate can be expressed by Equation (6), where (1 − α) represents the remaining portion of the reactive compound of the solid and n is the reaction order of pyrolysis.

(6) $$\frac{d\alpha }{dt}=Aexp\left(-\frac{E_{a}}{RT}\right){\left(1-\alpha \right)}^{n}$$

The summary equations of three isoconversional methods are listed in Table 5. If the activation energy is determined by the isoconversional method, the parameters A and n can be calculated using nonlinear least-squares fitting [83].

Three model-free non-isothermal methods can be used to extract activation energy from TGA data. Three typical methods, the Kissinger, Flynn–Wall–Ozawa method, and the Kissinger–Akahira–Sunose, were studied based on experimental tests at different heating rates [84].

(i) Kissinger method

This method evaluates the kinetics without calculating the activation energy in every conversion value. The equation is based on:

(7) $$ln\left(\frac{\beta }{{T^{2}}_{m}}\right)=ln\left(\frac{AR}{E_{a}}\right)-\frac{E_{a}}{RT_{m}}$$

Based on Equation (7), we can obtain the activation energy by plotting ln(β/T^2^_m_) versus 1000/T_m_ at different heating rates (β = dT/dt), where T_m_ is the temperature of the DTG peak. The activation energy is given by the slope of the plot, which equals −E_a_/R.

(ii) Flynn–Wall–Ozawa method (FWO)

This method calculates the activation energy and the preexponential factor without information about the reaction mechanism, which is only possible for one-step reaction calculations [83]. This method is based on the following equation:

(8) $$\ln\left(\beta_{i}\right)=ln\left(\frac{A_{\alpha }R}{R_{g}\left(\alpha \right)}\right)-5.331-1.052\frac{E_{\alpha }}{RT_{\alpha i}}$$

The activation energy is calculated by plotting ln β_i_ against 1000/T_αi_, where i denotes the heating value and conversion. The activation energy is calculated from the slope of the plot, which is equal to −1.052 E_α_/R.

(iii) Kissinger–Akihira–Sunose (KAS)

For non-isothermal TGA experiments at a linear heating rate, Equation (8) is changed to Equation (9)

(9) $$\frac{d\alpha }{f\left(\alpha \right)}=\frac{A}{\beta }exp\left(-\frac{E_{a}}{RT}\right)dT$$

Equation (9) from the initial condition of t = 0 at T = T_0_ will be obtained by integrating Equation (10).

(10) $$g\left(\alpha \right)\int_{0}^{\alpha }\frac{d\alpha }{f(\alpha )}=\frac{A}{\beta }\int_{T_{0}}^{T}\exp\left(-\frac{E_{a}}{RT}\right)dT=\frac{AE}{\beta R}p\left(\frac{E_{a}}{RT}\right)$$

The variables of A, f(α), and E_a_ are dependent on T, whereas E_a_ and A are independent of α, so Equation (10) can be further integrated into Equation (11)

(11) $$\ln g\left(\alpha \right)=\ln\left(\frac{AE_{a}}{R}\right)-ln\beta +lnp\left(\frac{E_{a}}{RT}\right)$$

The KAS method is based on the Coats–Redfern approximation, as shown in Equation (12) [85].

(12) $$p\left(\frac{E}{RT}\right)≅\frac{exp\left(-\frac{E_{a}}{RT}\right)}{{\left(\frac{E}{RT}\right)}^{2}}$$

And by combining Equation (7), this method is based on following equation:

(13) $$\ln\left(\frac{\beta_{i}}{{T^{2}}_{\alpha i}}\right)=ln\left(\frac{A_{\alpha }R}{E_{\alpha }g(\alpha )}\right)-\frac{E_{\alpha }}{RT_{\alpha i}}$$

The activation energy can be obtained by plotting ln β_i_ against 1000/T_αi_. The activation energy is calculated by the slope of the plot, which is equal to –E_a_/R.

Eimontas et al. reported catalytic pyrolysis experiments of PA6 waste fishing nets using a ZSM-5 zeolite catalyst (2.5–50 wt%) conducted with TGA coupled with Fourier-transform infrared spectroscopy and gas chromatography–mass spectrometry at various heating rates (5–30 °C/min). The kinetics of the ZSM-5-catalyzed pyrolysis were also investigated using model-free methods, including the KAS, FWO, and Friedman approaches [86]. Pannase et al. studied the decomposition of PA6 via slow pyrolysis, controlled by the contracting-sphere model. The activation energy reported via FWO, KAS, Vyzovkin, and Starink was nearly 205 kJ/mol. In contrast, the Friedman and Kissinger methods reported higher values of 238 and 228 kJ/mol, respectively [87].

#### 4.2.2. Glycolysis

Glycolysis is a widely used chemical recycling process for polyesters and polyurethanes, and it has also been investigated for PA6. It involves a nucleophilic substitution reaction, similar to transesterification or aminolysis, where glycol hydroxyl groups attack the ester or amide bonds in the polymer’s repeating units, resulting in chain scission and the formation of oligomeric products [75]. The glycolysis of PA6 in boiling ethylene glycol was among the earliest studied chemical degradation methods for PA6, producing oligoamides with amino and hydroxyl end groups. The catalysts examined in this process included zinc acetate, sodium glycolate, and poly(phosphoric acid) [88]. Hommez and Goethals studied the glycolysis of PA6 in the presence of phosphoric acid at 250 °C, yielding a mixture of low-molecular-weight compounds. The primary degradation products were ethylene glycol derivatives of CL and linear oligomers; no cyclic oligomers were detected. Two linear oligomers were identified: free carboxylic acid end groups and carboxylic acid end groups esterified with ethylene glycol [89]. Huczkowski and Kapko investigated the glycolysis of PA6 under pressure at 200–300 °C. They identified the optimal conditions for obtaining oligoamides with a number-average molecular weight of approximately 1000. The degradation process was effectively described within the experimental range studied using linear regression equations [90]. Zhao et al. introduced the first method for upcycling PA6 into high-performance polyamide derivatives. Their approach involves the initial degradation of PA6 into oligomers with reactive end groups through glycolysis. These oligomers act as intermediates in the synthesis of thermoplastic polyamide elastomers (TPAE), enabling direct integration with flexible polyethylene glycol oligomers without further degradation to monomers. By adjusting the reaction time and catalyst concentration, the molecular weight of the PA6 glycolysates can be controlled, thereby fine-tuning the mechanical properties of the resulting TPAE [91]. Kumar et al. reported that strong base catalysts such as cyclic amidines and sodium methoxide can effectively catalyze the glycolysis of PA6 and N-phenethyl 3-phenylpropanamide, exhibiting similar first-order rate constants (e.g., 1.22 × 10^5^ s^−1^), but also highlighted issues of catalyst deactivation and nonlinear dependence on catalyst loading [92].

#### 4.2.3. Ammonolysis

Ammonolysis typically refers to the reaction of ammonia with organic molecules, where the ammonia molecule splits into -H and -XH_2_ fragments. Simultaneously, the reacting organic molecule undergoes a double decomposition, splitting into two separate molecules. However, in some instances, the reacting organic molecule may retain portions of the ammonia molecule [58]. In ammonolysis, a PA6-containing material is heated in ammonia at 300–350 °C and at 500–2500 psig [29]. For each mole of the amide group, at least one mole of ammonia must be present in the batch or continuous reactor. The highest reported monomer yield reached 81 wt%, with water production and reaction equilibrium limiting further conversion. The primary by-products of this process include 5-cyanovaleramide, adiponitrile, 6-aminocaproamide, and aminocapronitrile [93]. Although these by-products can be further reacted to produce monomers, their separation is challenging, posing a significant disadvantage to the process.

Bordero et al. used a two-step ammonolysis depolymerization process for PA6 [94]. In the first step, PA6 was treated with n-butylamine at 300 °C and 45 atm, yielding hexamethylene diamine and N,N-dibutyl adipamide. In the second step, the N,N-dibutyl adipamide was subjected to ammonolysis at 285 °C and 50 atm. Under optimal conditions, the estimated yields were 48 wt% for adiponitrile and 100 wt% for hexamethylenediamine [95]. The yield of adiponitrile, which can either be sold as-is or converted to adipic acid or CL, was relatively low, indicating significant material loss. Long-chain polyamides are typically depolymerized through ammonolysis of secondary amide bonds into building blocks for new polymer synthesis. Coeck described a heterogeneous catalytic system based on Nb_2_O_5_ that operated at 200 °C, using cyclopentyl methyl ether as a green solvent and with limited additions of NH_3_ and H_2_ [96]. A simplified mechanism for the ammonolysis of nylon mixtures has been proposed, indicating that CL is present at higher concentrations than other major monomers in the batch reactor [97].

#### 4.2.4. Hydrolysis

Polyamides degrade through hydrolysis, leading to a decrease in molecular weight and mechanical strength and an increase in crystallinity over time, with temperature influencing the rate of these changes [98]. Hydrolysis is a decomposition process in which a bond within a compound is cleaved by adding hydrogen cations (H^+^) and hydroxide anions (OH^−^) from water. Hydrolysis has been used to depolymerize PA6 with acid or base catalysts [99]. High-pressure or superheated steam is commonly used in the uncatalyzed hydrolysis of PA6. The highest reported yield was 98 wt% for CL; however, the purity was not specified. In cases where purity was reported, it reached only 94.4 wt%, with lower yields, limiting the potential for high conversion during re-polymerization [100]. Although the yield from this process is high, non-PA6 components were not included in the reactor.

The presence of non-PA6 components in the reactor can reduce yield during hydrolysis. If this method were implemented industrially, additional purification steps would be required, thereby increasing capital and operating costs. Acid hydrolysis of PA6 uses an acid in water, often superheated steam, to decompose the polymer [101]. Acid hydrolysis is currently the only large-scale technique for depolymerizing post-industrial and post-consumer PA6. The primary acidic catalyst employed is orthophosphoric acid (H_3_PO_4_), a cost-effective chemical and strong dehydrating agent. Typically, 5–35 wt% of mineral acids combined with superheated steam produce ACA, a precursor to CL. Acid hydrolysis is generally conducted continuously, with operating temperatures ranging from 250 °C to 400 °C. The highest reported CL yield with this method was 96.4 wt% [102]. Gama et al. presented a method for the chemical recycling of PA6 waste using hydrochloric acid. Through solvolysis, PA6 was effectively depolymerized, and the influence of various reaction conditions on the depolymerization yield was investigated. The optimal conditions were 100 °C, a reaction time of 4 h, and an HCl/PA6 ratio of 11:1 (*wt*/*wt*) [103]. If complete hydrolysis of the monomeric products is achieved, the expected end product is ACA, the linear form of CL. Zhang et al. reported a one-pot catalytic process for degrading PA6 into ACA and CL using aqueous ammonia and a ruthenium (Ru) catalyst. By employing a ternary catalyst system comprising Ru/CeO_2_/RGO at 140 °C, a total yield of ACA and CL of up to 90.2 wt% was achieved [104].

The main disadvantage of acid hydrolysis is the need for multiple purification steps to obtain pure monomers, increasing process complexity and costs. Additionally, acid hydrolysis is unsuitable for non-mixed PA6 materials, further limiting its applicability [105], so using it with carpet waste would require near-perfect separation of the nylon fibers. Also, the CaCO_3_ from the carpet’s backing consumed some of the acid used [29]. As previously mentioned, determining the reaction mechanism in homogeneous kinetics is complex. Therefore, reaction models often estimate or rule out potential mechanisms [106]. However, basic principles of introductory chemistry can be applied to propose possible reaction mechanisms. Cyclization and crosslinking are generally not detected by TGA unless they occur alongside other decomposition mechanisms [107]. Some of the proposed mechanisms for the depolymerization and degradation of PA6 involve the formation of products such as alkenes, ammonia, CL, nitriles, tertiary amides (crosslinked structures), and water [108]. The characteristics of the predicted monomeric products closely match those of the water-soluble compounds generated after radiation exposure. McNeeley and Liu investigated three primary methods of PA6 depolymerization: liquid-phase hydrolysis, steam stripping, and solvent-free depolymerization. They also outlined the depolymerization process, purification methods, and waste management strategies required to obtain polymer-grade CL from crude CL produced during the depolymerization of PA6 waste [21]. Modern liquid-phase hydrolysis can achieve CL yields between 64 wt% and 96.4 wt%, depending on the catalyst and temperature, which typically ranges from 210 °C to 340 °C

Microwave heating surpasses conventional heating by achieving higher heating rates through direct molecular interaction, without heating the surrounding area. This results in faster material devolatilization, shorter conversion times, and shorter volatiles residence times, allowing quicker movement from internal hot zones to external cold regions. Consequently, secondary reactions of vapor-phase products are minimized, improving overall efficiency. Table 6 compares microwave and conventional heating.

Klun and Krž reported PA6 depolymerization using microwave energy for acid-catalyzed hydrolysis with phosphoric acid as the catalyst. A 200 W microwave was used for 12–23 min in a sealed reaction vessel. After 15 min, PA6 was completely solubilized. The resulting product mixture contained more than 90 wt% ACA and its linear oligomers, with a minor presence of cyclic products [109]. The microwave-assisted hydrolysis system can reach temperatures from room temperature up to 200 °C in only 10 min. In other words, the heating rate is up to 17 °C/min [13].

Microwave heating addresses the limitations of conventional treatments, such as their non-selectivity and the need for high temperatures. Tech-En Ltd. pioneered microwave techniques for the treatment of plastic waste in Hainault, UK. Their process involves mixing plastic-containing waste, referred to as plastic-containing transparencies, with a highly microwave-absorbent material, such as particulate carbon, to enhance heating efficiency [110].

**Figure 5:**
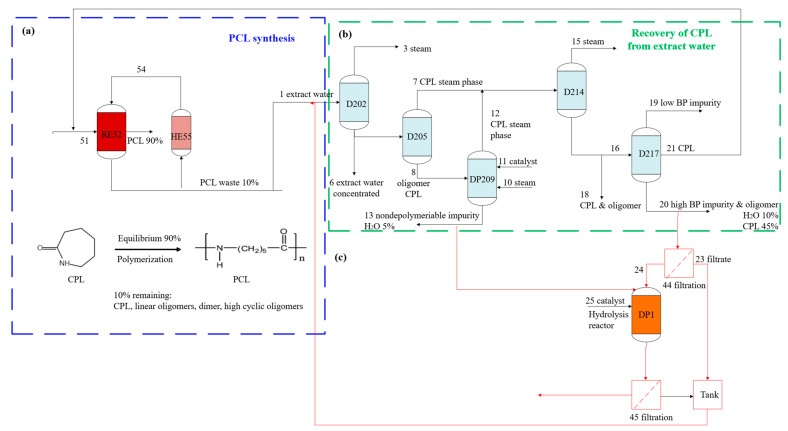
(**a**) PA6 polymerization process, (**b**) process recovery of CL, and (**c**) degradation of PA6 oligomers using hydrolysis, proposed by this study.

Chen et al. presented a closed-loop recycling of PA6 to CL using a phosphazene base, t-BuP4, as a green organocatalyst [111]. A closed-loop PA6 recycling process is illustrated in Figure 5. As shown in Figure 5a, approximately 10 mol% of the CL monomer remains unreacted during the polymerization of CL. Around 2.5 wt% of total cyclic oligomers are also formed, making it essential to recover and recycle these valuable raw materials. Recovered materials can be reused in subsequent polymerization reactions or repurposed for other applications. If left in the final PA6 product, residual monomers and oligomers can lead to undesirable effects during further processing, such as reduced material performance and stability [112].

A block diagram of the procedure is shown in (Figure 5b), as reported by Bormann et al. [3]. A distillation process (D202) receives the extracted water 1 produced during the extraction of PA6, as illustrated in Figure 5a. This distillation can be performed in a single or multiple steps. The extracted water from stream 1 is separated from the water in the form of stream 3. It can be reused after being recirculated via the extraction process. Concentrated extract water 4 is obtained as the bottom product and delivered to a subsequent distillation process (D205). The concentrated extract water is separated into a gaseous CL-steam phase 7 and a liquid oligomer-CL phase 8 using this distillation process (D205). The concentrated CL extract water is separated into a gaseous CL-steam phase 7 and a liquid oligomer-CL phase 8 by this distillation process 5. Steam strips the CL from the oligomer-CL phase and sends it to a subsequent distillation process 14 with the CL-water phase 7, as illustrated in stream 12. The oligomer-CL elements that are not depolymerizable in process 9 are discharged as bottom product 13 (WS-13) and discarded as waste. The slurry waste stream 20 will be filtered; the filtrate will flow into stream 23, and the solid waste will be collected as a cake in stream 24 (WS-24). The WS-24 and WS-13 feed streams to the reactor will be hydrolyzed with an acidic solution [14] (Figure 5c). Monomer yield analysis revealed distinct substrate preferences: WS-13 achieved a balanced recovery of ACA and CL, while WS-24 produced mainly ACA. Morphological and thermal stability studies further supported these trends, confirming that porosity and lower crystallinity promote efficient acid penetration and chain scission. The resulting depolymerized product will be filtered and pumped to stream 1 in the CL recovery process (Figure 5b).

#### 4.2.5. Degradation Kinetics by the Hydrolytic Method

The amounts of PA6 reacted and unreacted were determined. The kinetics of PA6 depolymerization can be thought of as a pseudo-first-order reaction. As a result, the equations followed:

(14) $$-\frac{d[\mathrm{PA}6]}{dt}={k[\mathrm{PA}6]}^{m}\;{\left[\mathrm{acid}\right]}^{n}$$

If the reaction is a pseudo-first-order reaction, the reaction expression is given in Equation (15):

(15) $$-\frac{d[\mathrm{PA}6]}{dt}=k^{\prime }[\mathrm{PA}6]$$

By integrating Equation (15), Equation (16) is obtained:

(16) $$\ln\;\frac{{\left[\mathrm{PA}6\right]}_{0}}{{\left[\mathrm{PA}6\right]}_{t}}=k^{\prime }\cdot t$$

As PA6 is solid, it is solid. Thus, the amount can be expressed as weight. Therefore, the expression of $\frac{{\left[\mathrm{PA}6\right]}_{0}}{{\left[\mathrm{PA}6\right]}_{t}}$ can be expressed as α/(α − x) where α and α − x are initial weights at some time interval, respectively. Thus, Equation (16) can be changed as follows:

(17) $$\ln\;\frac{\alpha }{\alpha \;-\;x}=k^{\prime }\cdot t$$

The relation between ln (α/(α − x)) and reaction time. The slope of the plot gives the reaction rate constant for hydrolysis.

#### 4.2.6. Biological Treatment Practices for CL Waste

Biological recycling, or biorecycling, involves aerobic and anaerobic processes in which microorganisms, such as bacteria and fungi, and enzymes break down materials into smaller fragments or lower-molecular-weight compounds [70,113]. Enzymes have shown potential to degrade high-molecular-weight polyamides, enabling polyamide recovery under mild conditions. Friedrich et al. demonstrated that white rot Basidiomycetes fungi could degrade PA6 when cultured with PA6 as the sole nitrogen source. Over 60 days, they observed a 67 wt% reduction in PA6 molecular weight from 16.9 kDa to 4.5 kDa [114]. Bell et al. screened 40 potential natural and engineered nylon-hydrolyzing enzymes (nylonases) using mass spectrometry to quantify eight compounds produced by enzymatic hydrolysis of PA6. Comparative time-course reactions conducted at temperatures ranging from 40 to 70 °C demonstrated enzyme-dependent variations in product distributions and the extent of PA6 film depolymerization. The most active nylonase, a thermostabilized NylCK variant (an N-terminal nucleophile (Ntn) hydrolase, NylCK-TS, with a T_m_ of 87.4 °C, 16.4 °C higher than the wild-type), hydrolyzed 0.67 wt% of a PA6 film [115]. Turk et al. presented biosynthetic pathways starting with 2-oxoglutarate that incorporate bioconversions from the ketoacid elongation pathway known in methanogenic archaea, for implementation in *E. coli*. They achieved ACA production levels up to 160 mg/L in lab-scale batch fermentations. Furthermore, the total amount of ACA and related intermediates generated by this pathway exceeded 2 g/L in lab-scale fed-batch fermentations [116].

#### 4.2.7. Hydrothermal

Khuntia et al. studied the depolymerization of PA6 using the conventional hydrothermal method, which employs various organosulfonic acids, including methane sulfonic acid, para-toluene sulfonic acid, benzene sulfonic acid, and tetra-butyl ammonium bromide (TBAB) as a phase-transfer catalyst. Thermodynamic parameters, including enthalpy, heat, and reaction entropy, were evaluated using the Eyring–Polanyi equation. The combined catalytic effect of organosulfonic acids and phase-transfer catalysts provides an environmentally friendly method for effectively depolymerizing PA6 [50].

Selective hydrolysis of PA6 and polyamide-66 from commercial multicomponent PA6/polyamide-66/polypropylene carpets was demonstrated using a microwave-assisted acid-catalyzed hydrothermal process, which yielded monomeric products and solid polypropylene residue. The optimized hydrochloric acid-catalyzed process selectively produced monomers, such as 6-hydroxyhexanoic acid (ACA) and adipic acid, and hexamethylenediamine within 30 min. Wang et al. investigated the dissolution, crystallization, and hydrolysis behaviors of PA6 in superheated water (140 °C ≤ T ≤ 200 °C). Hydrolysis became more pronounced at higher temperatures and longer processing times, as PA6 dissolved in superheated water [13].

The depolymerization reaction is consecutive, and the following Equation describes the decomposition of PA6.

(18) $$PCL\overset{k_{1}}{\to }ACA\overset{k_{2}}{\to }CL$$

The production rates of ACA and CL are given by Equations (19) and (20).

(19) $$\frac{dC_{ACA}}{dt}=k_{1}C_{PCL}-k_{2}C_{ACA}$$

(20) $$\frac{dC_{CL}}{dt}=k_{2}C_{ACA}$$

The values of k_1_ and k_2_ were determined by comparing the observed time variation in *C_CL_*, *C_ACA_*, and *C_CL_*. Iwaya et al. present a result of s. The values determined are k_1_ = 5.5 × 10^−4^ s^−1^ and k_2_ = 1.0 × 10^−2^ s^−1^ at 573 K, 25 Mpa [38].

Meng et al. investigated the macroscopic phase transitions and microscopic hydrolytic mechanisms of PA6 under milder reaction conditions (240–280 °C). Under optimized conditions (280 °C, 2.5 h, and a bath ratio of m(H_2_O)/m(PA6) = 9), the conversion of CL reached up to 94.0 wt%. The hydrolytic process of PA6 can be divided into four stages: the annealing stage, the intermediates formation stage, the transition stage, and the monomerization stage. Kinetic analysis used simple first-order and tandem reaction models [117]. Zhang et al. proposed a novel hydrothermal clean-water depolymerization method to recover high-purity monomers from PA6, leveraging the reversibility and hydrophilicity of amide bonds via hydrothermal amphoteric hydrolysis. Following this process, repolymerization of the monomer was completed, and new PA6 was generated via one-pot solution polycondensation, thereby achieving a closed-loop recycling process [118]. Dos Passos et al. investigated the optimization of hydrothermal liquefaction for the chemical recycling of PA6 and polyethylene multilayer plastic films. A two-step process was employed, using subcritical water to selectively depolymerize PA6 into CL. Experiments with raw PA6 yielded up to 88 wt% CL under optimized conditions (325 °C, 30 min). In the two-step process, CL recovery from multilayer films approached 100 wt% [119].

#### 4.2.8. Monomer Design Strategy

Cheung et al. described the chemical recycling of CL, focusing on depolymerization and polymerization processes. The concept involves zinc-catalyzed methanolysis to depolymerize PA6 and produce methyl 6-hydroxyhexanoate, which is then polymerized via a zinc-catalyzed condensation to regenerate new PA6, effectively closing the recycling cycle. Specifically, with zinc(II) acetate as the catalyst, PA6 was depolymerized at turnover frequencies of up to 468 h^−1^ using microwave heating [120].

Alberti et al. reported a process for depolymerizing end-of-life PA6 via ring-closing reactions to produce the building block N-acetylcaprolactam. By using a combination of acetic anhydride as a depolymerization reagent and catalytic amounts of 4-dimethylaminopyridine under microwave irradiation, the process was completed in a short time (15 min). The resulting product, N-acetylcaprolactam, was then converted to CL by transferring the acetyl group to 2-aminoethanol, forming N-(hydroxyethyl)acetamide, which serves as a precursor to poly(N-vinylacetamide) [121].

The hydrolytic process for PA6 can achieve a maximum CL yield of 94.0 wt% under optimized conditions, and a novel recycling protocol from PA6 to regenerated PA6 has been proposed, demonstrating significant energy savings and comparable properties to commercial PA6 [117].

#### 4.2.9. Ionic Liquid

Using ionic liquids offers a significant advantage, as the monomer can be directly recovered by distillation from the reaction mixture. Kamimura et al. studied the efficient depolymerization of PA6 in hydrophilic ionic liquids under microwave irradiation at 300 °C. Depolymerization was complete within 60 min. A simple extraction procedure easily separated CL, and the ionic liquids were recovered and reused several times. Additionally, the inclusion of catalytic amounts of N,N-dimethylamino pyridine effectively promoted the depolymerization process. This improved method avoids energy-intensive direct distillation typically required to separate CL from ionic liquids [46,122]. Wursthorn et al. demonstrated a catalytic system based on readily accessible lanthanide trisamides, which catalyze the rapid and selective depolymerization of PA6. The process is solvent-free, highly selective, and operates under mild conditions, offering a high-yield method for depolymerizing PA6 [123]. Yuan et al. studied that PA6 was degraded using hydrochloric acid to produce a “pseudo” amino acid ionic liquid ε-aminocaproic acid hydrochloride. This degradation process offers the advantages of operational simplicity and mild reaction conditions, while avoiding an energy-consuming and tedious purification process [47]. Product separation is often challenging in ionic-liquid-based PA6 depolymerization, prompting research into solvent-free catalytic methods at elevated temperatures (~523 K or higher) [40,124].

#### 4.2.10. Reactor Design Considerations for PA6 Chemical Recycling

Hydrolysis depolymerization of PA6 to CL is a promising reprocessing method that delivers high monomer yields (>90 wt%) and works well with post-consumer waste after additive separation. This section explains the applications and benefits of four reactor types: Continuous Stirred-Tank Reactor (CSTR), Fluidized-Bed/Vertical Stripping Reactor, Extruder/Tubular Reactor, and Microwave Reactor. Each uses different engineering principles to improve reaction speed, energy efficiency, and scalability for technologists aiming for circular PA6 recycling.

(i) Continuous Stirred-Tank Reactors (CSTRs) or Baffled Variants. CSTRs operate in liquid-phase hydrolysis at temperatures of 250–330 °C and pressures of 2–20 bar, using excess water (10:1 *w*/*w* ratio). They employ mechanical agitation or baffles to maintain a homogeneous molten PA6-water-catalyst slurry. Injecting superheated steam improves mass transfer, breaking amide bonds through acid-catalyzed hydrolysis (e.g., H_3_PO_4_ at 5–10 wt%). Applications include industrial-scale processing of pre-separated textile wastes or industrial scrap, where consistent feeding via pumps supports continuous operation. Key advantages are uniform temperature control (±2 °C), which helps prevent hotspots that can degrade oligomers, and high throughput (10–100 t/day). Residence times of 1–4 h can produce 75–96 wt% caprolactam, with recycle streams for water and catalyst decreasing costs by 20–30 wt% compared to batch systems. For technologists, CSTRs are effective at handling variable impurity loads after solvent extraction, as their robust mixing prevents fouling—crucial for achieving >94 wt% yields from mixed PA6 sources. Energy integration through heat recovery from overhead vapors further enhances efficiency, making CSTRs ideal for mid-scale plants [125].
(ii) Fluidized-Bed or Vertical Stripping Reactors. These processes use steam stripping at 260–350 °C and 0.6–1.5 bar, with countercurrent superheated steam (ratios of 2–17:1) to fluidize PA6 particles or melt while stripping water-soluble caprolactam vapor overhead. Vertical designs, such as DSM/Antron’s, incorporate multi-stage trays to promote plug-flow behavior and minimize backmixing. Primary applications target post-carpet or composite wastes, where inorganic fillers (such as glass fibers) pass through as residue. Benefits include over 95 wt% yields with phosphoric acid catalysts, short residence times (0.5–2 h), and the natural volatilization of impurities during stripping—reducing downstream distillation by 15–25 wt%. Fluidization ensures excellent heat and mass transfer (kLa > 0.1 s^−1^), preventing char formation from flame retardants, while modular stacking scales capacity to over 50 tons per day. Experts value the energy efficiency: steam acts as both reactant and stripping agent, with exergy losses below 40 wt% through integration. Proven in plants from the 1990s recovering 30 kt/year of caprolactam, these reactors are preferred for their robustness and low-maintenance operation on dirty feeds [21].
(iii) Extruder/Pre-Melter + Tubular Reactors. Solvent-free hydrolysis employs twin-screw extruders (220–300 °C, 0.013–2 kPa vacuum) to pre-melt and devolatilize PA6, which then feeds into wiped-film or tubular reactors for thin-film hydrolysis. Lanthanide catalysts (e.g., La-Organic frameworks) speed up reactions under mild conditions. The focus is on producing clean, separated composites or films, enabling small footprints for on-site recycling. Benefits include the fastest kinetics (over 90 wt% conversion in less than 30 min), the lowest energy consumption (50–70 kWh/t compared to over 100 for steam), and minimal water use (<1:1 ratio), reducing effluent by 80 wt%. Wiped-film systems auto-clean and tolerate 1–5 wt% additives without clogging, while overhead vacuum removes impurities such as plasticizers. This method provides over 99 wt% monomer purity directly, eliminating multi-stage distillation, and reduces CAPEX by 30% for plants processing 5–20 tons per day. Emerging pilot projects emphasize modularity for decentralized waste management [21,125].
(iv) Microwave Reactors. Microwave-assisted hydrolysis (150–250 °C, 5–30 min) uses dielectric heating to selectively excite water-PA6 interfaces, increasing rates 5–10 times through hotspots and pressure build-up in sealed vessels. Batch or semi-continuous setups using SiC susceptors handle small-scale (1–10 kg/batch) post-textile waste. These applications are suitable for R&D or specialized recycling of dyed PA6, where rapid energy delivery helps degrade colorants. Benefits include very short processing times (yielding over 92 wt% compared to 4 h with conventional methods) and 40 mol% for catalyst optimization. Unlike convective heating, microwaves penetrate deeply (wavelength ~2.8 cm at 2.45 GHz), enabling uniform depolymerization even in thick feeds. Challenges such as scale-up and hotspot formation are addressed using continuous-flow methods with screw feeders. Technologists benefit from easy integration with pre-separation processes (e.g., glycol dissolution), producing low-oligomer crude (<2 wt%) that simplifies purification [126].

### 4.3. Techno-Economic and Carbon Intensity Assessment of PA6 Chemical Recycling

The industrial feasibility of PA6 chemical recycling must be assessed using an integrated techno-economic and life-cycle framework rather than reaction yield alone [11,127]. Although laboratory studies frequently report CL yields above 90 wt%, overall economic performance is governed by energy demand, separation intensity, catalyst cost, and system integration. Minor et al. developed an Aspen Plus–Python process model (https://github.com/A-JMinor/Python-Aspen-Plus-Connected-Model-for-the-Calculation-of-Equipment-Costs, accessed on 1 January 2026) that compares alcoholysis and hydrolysis routes, demonstrating that acidic hydrolysis offers the most favorable balance between reaction performance and process simplicity. However, multi-stage distillation, required for polymer-grade CL (>99 wt%), dominates operating expenditure and strongly influences the minimum selling price [11].

Capital expenditure remains higher than mechanical recycling due to high-temperature reactors (250–400 °C), corrosion-resistant materials, and solvent recovery systems. Continuous hydrolysis configurations partially mitigate capital intensity through scale economies, yet separation and wastewater treatment remain cost drivers. Operating costs are primarily determined by thermal energy consumption, acid or catalyst usage, and purification duty. Microwave-assisted systems may reduce reaction time and thermal residence time, but their economic benefits depend on electricity prices and scale efficiency.

From a carbon-intensity perspective, chemical recycling may offset emissions from virgin CL production when heat integration and renewable energy sources are employed. Tonsi et al. conducted a life-cycle assessment comparing selective dissolution, mechanical, and chemical recycling routes, highlighting that environmental performance is highly sensitive to the energy source and solvent recovery efficiency. When fossil-based heat is used, greenhouse gas emissions may approach those of virgin production; conversely, integrating renewable energy significantly improves carbon performance [2]. According to Tonsi et al.’s report [128], the life cycle assessment of polyamide selective dissolution recycling was reported, analyzing different process configuration scenarios in which the precipitation of PA6 is induced either by the addition of an antisolvent or by solvent evaporation, and also exploring the possibility of valorizing the non-nylon-coupled material. A comparison of mechanical, chemical, and solvent-based recycling is made with respect to applicability and environmental impacts, with all calculations accounting for renewable energy sources [128]. However, as noted by McNeeley and Liu (2024), the ultimate carbon intensity is highly dependent on the efficiency of the monomer purification stage, which remains the primary energy consumer in the depolymerization process [21]. The comparative assessment of virgin vs. recycled PA6 is listed in Table 7.

Given that the textile industry accounts for roughly 10% of global carbon emissions, the rapid hydrolysis of waste PA6 textiles into CL is a frontier for reducing carbon intensity [21]. Overall, acidic hydrolysis currently represents the only pathway approaching near-term techno-economic viability, if separation energy demand and carbon intensity are minimized through process intensification and integrated reaction–separation design. Future progress will depend more on system-level optimization and decarbonized energy inputs than on incremental yield enhancement alone. Minor et al. developed an automated Python-Aspen Plus-LCA workflow to analyze compositional variations in PA6 waste. Results for the hydrothermal route depend on the water-to-feed ratio, but its GWP remains higher than that of fossil-based CL. Alcoholysis and acidic processes reduce GWP by about 35%, while the NaOH route lowers it to 1.46 kg CO_2_-eq/kg CL, a reduction of 80 wt%. Since the NaOH process shows the best environmental and economic results, future studies should focus on scaling up solvent-free chemical recycling [129].

### 4.4. Effect of Additives on PA6 Depolymerization and Caprolactam Purity

In real post-consumer and post-industrial PA6 waste streams, additives such as thermal stabilizers, flame retardants, plasticizers, pigments, and processing aids are commonly present (Table 8). These components can significantly influence depolymerization reactions, affect CL yield and purity, and therefore pose a key challenge for industrial-scale recycling. Thermal and oxidative stabilizers, often based on hindered phenols or phosphites, can inhibit chain scission reactions by scavenging radicals, thereby slowing depolymerization kinetics, especially in pyrolysis and catalytic systems. In contrast, under hydrolytic conditions, their impact is less significant but may still contribute to residue formation and minor contamination in the liquid phase. Flame retardants, particularly brominated or phosphorus-based compounds, create more serious problems. During thermal processes like pyrolysis, these additives may decompose into halogenated or phosphorus-containing by-products, which can contaminate the recovered monomer stream and increase corrosion and environmental risks. In hydrolysis-based systems, inorganic flame-retardant residues (such as aluminum hydroxide) may remain as solids, interfering with heat transfer and reactor operation.

Plasticizers and low-molecular-weight additives can volatilize or dissolve during depolymerization, leading to co-distillation with caprolactam and complicating purification. Their presence increases the burden on downstream separation, particularly in achieving polymer-grade CL (>99 wt%). Similarly, dyes and pigments, especially those containing metal complexes, can introduce trace metal impurities that affect catalyst performance in repolymerization and may require additional purification steps such as adsorption or ion exchange. Purification techniques effectively remove approximately 1 wt% impurities from crude CL by converting reactive contaminants (e.g., glycols, amines, acids) into higher-boiling species or leveraging volatility differences, achieving >99% purity [130]. Vacuum distillation and chemical treatment are standard for handling 1 wt% levels, often in multi-stage setups for industrial recycling of PA6 using water [131] or phosphorus pentoxide [130]. Follow P_2_O_5_ treatment with Ca(OH)_2_ neutralization and final distillation to eliminate acids/color; monitor via GC-MS. These yield > 94 wt% from PA6 streams, enabling closed-loop recycling [21].

**Table 8:** Effect of typical PA6 additives on depolymerization performance and monomer purity.

Additive Type	Typical Compounds	Effect on Depolymerization	Impact on CL Selectivity	Impact on Product Purity	Process Implication	Ref.
Thermal stabilizers	Hindered phenols, phosphites	Radical scavenging inhibits chain scission, especially in thermal processes	Slight decrease	Minor organic residues	Slower kinetics; residue accumulation	[132,133]
Flame retardants (halogenated)	Brominated FRs	Decomposes to halogenated volatiles during pyrolysis	Moderate decrease	Significant contamination (toxic/halogenated species)	Requires gas scrubbing and advanced purification	[9,134,135]
Flame retardants (inorganic)	Al(OH)_3_, Mg(OH)_2_	Remain as solid residues; alter heat transfer	Minimal	Low (solid-phase impurities)	Reactor fouling; filtration required	[136]
Plasticizers/low-MW additives	Phthalates, adipates	Volatilize or dissolve; may co-distill with CL	Slight decrease	Moderate contamination	Increased separation load (distillation)	[137]
Dyes/pigments	Organic dyes, metal-complex pigments	Mostly inert; possible decomposition or metal release	Negligible	Trace metals/color impurities	Requires adsorption or ion-exchange polishing	[138]
Fillers/reinforcements	Glass fibers, CaCO_3_	Inert; affects mixing and heat transfer	None	Low (solid separation needed)	Mechanical separation and filtration are required	[139]

### 4.5. Decision Framework for PA6 Recycling Pathway Selection

**Figure 6:**
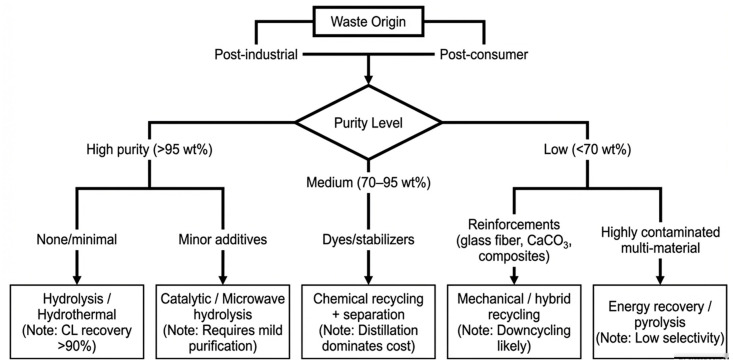
Decision framework for selecting PA6 recycling pathways based on waste characteristics.

To facilitate the translation of PA6 recycling technologies from laboratory research to industrial implementation, a decision framework is proposed to systematically match waste characteristics with appropriate recycling pathways (Figure 6). This framework integrates key factors, including material purity and the presence of additives or fillers, which critically influence process feasibility, efficiency, and economic performance.

Waste origin serves as the primary classification criterion. Post-industrial PA6 waste typically exhibits high purity and uniform composition, making it well-suited for chemical recycling routes such as hydrolysis or hydrothermal depolymerization, where high caprolactam yields can be achieved. In contrast, post-consumer waste streams are more heterogeneous, often containing dyes, mixed polymers, and inorganic fillers, which complicate reaction pathways and downstream purification.

Material purity further determines process selection. High-purity PA6 enables direct chemical depolymerization with minimal pre-treatment. However, as impurity levels increase, additional separation steps become necessary, particularly in achieving polymer-grade caprolactam. These purification processes, especially distillation, are known to account for the majority of energy consumption and operational costs in PA6 recycling systems. Consequently, medium- to low-purity streams may favor hybrid or mechanical recycling approaches depending on economic constraints.

The presence of fillers and multi-material structures introduces additional limitations. Components such as CaCO_3_ or reinforcing fibers can interfere with catalytic reactions and reduce depolymerization efficiency, making mechanical recycling or energy recovery more practical alternatives for highly contaminated waste streams. Overall, this framework highlights that no single recycling technology is universally optimal. Instead, effective PA6 recycling requires a feedstock-driven strategy that integrates material characteristics with process selection, emphasizing the importance of sorting, pre-treatment, and system-level optimization to achieve sustainable, economically viable circularity.

## 5. Conclusions

This review systematically compared PA6 recycling technologies by integrating reaction performance, process conditions, and industrial constraints. Hydrolysis-based depolymerization consistently achieves CL yields of 64 to 96.4 wt%, with optimal operation typically occurring at 250 to 340 °C under acidic or hydrothermal conditions. Microwave-assisted hydrolysis further reduces reaction times from several hours to minutes while maintaining comparable monomer yields (>90 wt%), indicating clear advantages in process intensification and energy efficiency. However, these benefits are partially offset by the energy demand associated with downstream purification, particularly multi-stage distillation required to achieve polymer-grade CL (>99 wt%), which remains the dominant contributor to operating cost.

Pyrolysis-based processes can reach CL yields above 85 wt% under catalytic conditions (330–360 °C), but product selectivity is highly sensitive to catalyst type and reaction environment, often leading to broader product distributions and additional separation requirements. Glycolysis and ammonolysis provide alternative depolymerization routes but are inherently limited by incomplete monomer recovery, formation of side products, and complex downstream purification, restricting their applicability for closed-loop recycling. Ionic-liquid-assisted systems and homogeneous catalytic depolymerization demonstrate high selectivity under milder conditions; however, their scalability is constrained by challenges in solvent recovery, catalyst cost, and system complexity.

From an industrial perspective, the feasibility of PA6 chemical recycling is governed less by depolymerization yield and more by process integration, particularly the coupling of reaction and separation steps. Current evidence indicates that processes capable of integrating depolymerization with efficient monomer recovery and purification, such as continuous hydrolysis systems integrated with heat, offer the most realistic pathway toward industrial implementation. In contrast, approaches requiring extensive solvent handling, multi-step reaction schemes, or highly sensitive catalysts remain at the laboratory or pilot scale.

## Figures and Tables

**Table 1:** Advantages and disadvantages of different PA6 recycling methods.

Method	Summary	Advantages	Disadvantages	Ref.
Incineration	High-temperature burning.	Recovery energy. Relatively mature energy.	Produce poisonous gas. Serious pollution and public hazard. The installation is expensive.	[41]
Mechanical	Only change the physical form, such as the packaging of raw materials, for reuse.	Simple operation, less pollutant efficiency. Relatively less equipment investment.	Certain requirements for wastes. Product performance reduced the range. Low economic benefit. Products from recycled waste may not be durable.	[8]
Chemical	Degradation reaction to lower molecular weight.	Get pure material monomer. The product can be used as a raw material for the product or to prepare new products.	High temperature and high pressure (cost-intensive).	[42]

**Table 2:** Summary of the chemical recycling of PA6 waste.

Material	Product (%)	Method	Catalyst	T (°C)	Time	Solid:Solution	Ref.
PA6	77.9 wt% CL	hydrolysis	HPA	330	85 min	1:15	[43]
PA6	55 wt% oligomer	hydrogenative	Ruthenium	150	48 h	1:2.5	[44]
Teabag waste	59.2 wt% CL	pyrolysis	-	700	-	-	[45]
PA6 plastic	86 wt% CL	ionic liquids	PP13 TFSI	300	6 h	-	[46]
PA6	85 wt% ACA	ionic liquids	HCl, 30 wt%	109	24 h	-	[47]
PA6 fiber	93 wt% ACA	hydrolysis	HCl, 30 wt%	90	4 h	1:25	[48]
PA6	85 wt% CL	hydrothermal	-	360	60 min	-	[38]
PA6	78 wt% CL	Sub-critical water	HPA	300	85 min		[43]
PA6	62.3 wt% CL	Hydrolysis	Zeolite Hβ-25	345	30 min	1:10	[49]
PA6 carpet waste	monomeric	Hydrolysis	HCl 10 wt%	200	3 h	1:20	[13]
PA6	96 wt%, unidentified	Hydrolysis	toluene sulfonic acid PTC	100	10 h	1:6	[50]

**Table 3:** Comparative assessment of PA6 recycling technologies toward CL recovery.

Recycling Route	Temp (°C)	CL Yield (wt%)	Reaction Time	Activation Energy (kJ/mol)	Separation Complexity	Energy Intensity	Carbon Reduction Potential	Industrial Feasibility
Mechanical recycling	220–260	N/A	Minutes	N/A	Low	Low	Moderate (50–70%)	High (Downcycling)
Acid hydrolysis	250–400	85–96	1–4 h	160–220	High (distillation-heavy)	Moderate	High (30–50%)	High
Hydrothermal (Subcritical Water)	240–325	80–94	0.5–2 h	~180–210	Moderate–High	Moderate	High	Medium–high
Microwave-Assisted Hydrolysis	200–300	>90	<30 min	-	Moderate	Moderate (electricity-based)	High (if renewable electricity)	Medium
Catalytic Solvent-Free Depolymerization	220–260	≥99 (lab scale)	<2 h	-	Moderate	Low–Moderate	Potentially high	Low–medium (scale challenge)
Pyrolysis	330–800	70–90	1–4 h	~200–240	Low	High	Low–moderate	Medium
Glycolysis/alcoholysis	200–300	Oligomer-dominant	2–10 h	Variable	High	Moderate	Moderate	low–medium
Ionic liquid-assisted	250–300	85–95	1–2 h	-	High (solvent recovery)	Moderate–High	Uncertain	Low
Biological/enzymatic	40–70	<1% film conversion	Days–Weeks	Enzymatic	Low	Very Low	Very high (theoretical)	Academic Stage
Energy recovery (Incineration)	>800	0	Immediate	N/A	Very Low	Very High	Negative	Mature but non-circular

**Table 4:** Representative industrial enterprises for PA6 recycling and caprolactam recovery.

Company	Year of Launch	Technology	Final Product	Processing Capacity	Ref.
Aquafil (ECONYL^®^), Arco, Italy.	2011	Hydrolysis-based depolymerization	CL → regenerated PA6	35,000 ton/year	[59]
DOMO Chemicals (TECHNYL^®^ 4EARTH^®^), Leuna, Germany	2018	Chemical recycling (hydrolysis + purification)	Virgin-grade PA6	Not publicly disclosed	[60]
BASF (ChemCycling™), Ludwigshafen, Germany.	2018	Pyrolysis-based feedstock recycling	Secondary raw materials for polymers (incl. PA)	Pilot → industrial scale	[61]
Toray Industries, Tokyo, Japan	2010	Depolymerization and purification	CL/Nylon 6	Demonstration scale	[62]
RadiciGroup, Bergamo, Italy	2018	Mechanical + chemical recycling	Engineering PA6 materials	Industrial scale	[63]

**Table 5:** Summary of the isoconversional method.

Method	Expression	Plots	Slope Value
Kissinger	$ln\left(\frac{\beta }{{T^{2}}_{m}}\right)=ln\left(\frac{AR}{E_{a}}\right)-\frac{E_{a}}{RT_{m}}$	$ln\left(\frac{\beta }{{T^{2}}_{m}}\right)\;versus\;1/T$	−E_a_/R
Kissinger–Akahira–Sunose	$\ln\left(\frac{\beta_{i}}{{T^{2}}_{\alpha i}}\right)=ln\left(\frac{A_{\alpha }R}{E_{\alpha }g(\alpha )}\right)-\frac{E_{\alpha }}{RT_{\alpha i}}$	ln $\left(\frac{\beta }{{T^{2}}_{m}}\right)$ versus 1/T	−E_a_/R
Flynn–Wall–Ozzawa	$\ln\left(\beta_{i}\right)=ln\left(\frac{A_{\alpha }R}{R_{g}\left(\alpha \right)}\right)-5.331-1.052\frac{E_{\alpha }}{RT_{\alpha i}}$	ln β versus 1/T	−1.0516 E_a_/R

**Table 6:** Summary of microwave and conventional heating.

Microwave Heating	Conventional Heating
Inverse thermal conventional de-outside	Thermal gradient (outside–inside)
Gradient	Conduction and convection currents
Very short and instant heating	Longer processing times
No or lower solvent is possible.	No or lower solvent savings
Higher product quality and quantity are possible	Product quality and quantity can be quantified.
Moderate to low consumption and quantity of energy	High energy consumption
Straightforward process configuration	Simple process configuration

**Table 7:** Comparative assessment: virgin vs. recycled PA6.

Metric	Virgin PA6 (Conventional)	Chemically Recycled PA6
Primary feedstock	Crude oil/benzene	Post-consumer/industrial waste
Energy consumption	High (synthesis + polymerization)	Moderate (depolymerization + purification)
GWP (kg CO_2_-eq)	8.0–9.5	1.5–4.5 (Reduction of ~50–70 wt%)
Technical quality	Standard grade	Near-virgin (high purity CL)
Economic barrier	Market volatility of oil	Cost of collection & sorting

## Data Availability

No new data were created or analyzed in this study. Data sharing is not applicable to this article.

## Abbreviations

ACA	6-Aminocaproic acid
CL	Caprolactam
CAPEX	Capital expenditure
E_a_	Activation energy
HPA	Phosphotungstic heteropoly acid
PTC	Phase-transfer catalyst

## References

[1] Hirschberg V., Rodrigue D. (2023). Recycling of polyamides: Processes and conditions. J. Polym. Sci..

[2] Tonsi G., Maesani C., Alini S., Ortenzi M.A., Pirola C. (2023). Nylon recycling processes: A brief overview. Chem. Eng. Trans..

[3] Bormann A., Albrecht M., Samlitschka F. (2017). Process for the Recovery of ε-Caprolactam from Extract Water. U.S. Patent.

[4] Damayanti D., Wulandari L.A., Bagaskoro A., Rianjanu A., Wu H.S. (2021). Possibility Routes for Textile Recycling Technology. Polymer.

[5] Hay J.N., Booth A. (1972). The effect of a secondary process on the course of polymer crystallisation. Br. Polym. J..

[6] Reimschuessel H.K. (1977). Nylon 6. Chemistry and mechanisms. J. Polym. Sci. Macromol. Rev..

[7] Zhang S., Wu Y., Ji P., Ran Q., Wang H., Chen B., Wang C. (2023). Sustainable Production of Polyamide 6 Fibers: Direct Melt Spinning and Efficient Reuse of Residual Oligomers during Polymerization. ACS Sustain. Chem. Eng..

[8] Schyns Z.O.G., Shaver M.P. (2021). Mechanical Recycling of Packaging Plastics: A Review. Macromol. Rapid Commun..

[9] Dogu O., Pelucchi M., Van de Vijver R., Van Steenberge P.H.M., D’Hooge D.R., Cuoci A., Mehl M., Frassoldati A., Faravelli T., Van Geem K.M. (2021). The chemistry of chemical recycling of solid plastic waste via pyrolysis and gasification: State-of-the-art, challenges, and future directions. Prog. Energy Combust. Sci..

[10] Feng Y., Quan X., Wang Q., Zhang Y., Liu C., Yuan X., Zhao S., Yang J., He W., Guo K. (2025). Recent Advances in the Chemical Recycling of Polyamide for a Sustainable Circular Economy. Ind. Eng. Chem. Res..

[11] Minor A.-J., Goldhahn R., Rihko-Struckmann L., Sundmacher K. (2023). Chemical Recycling Processes of Nylon 6 to Caprolactam: Review and Techno-Economic Assessment. Chem. Eng. J..

[12] Clark R.A., Shaver M.P. (2024). Depolymerization within a Circular Plastics System. Chem. Rev..

[13] Bäckström E., Odelius K., Hakkarainen M. (2021). Microwave Assisted Selective Hydrolysis of Polyamides from Multicomponent Carpet Waste. Glob. Chall..

[14] Pristiani M., Damayanti D., Wu H.-S. (2025). Microwave-Assisted Acid Hydrolysis of PA6 Wastes in PA6 Process: Kinetics, Activation Energies, and Monomer Recovery. Processes.

[15] Barr M.L., Cai H., Esposito A., Freundlich J., King D.W., Mendolia M., Moghe B., Petroff L.J., Schamper T., Skinner M.W. (2002). Cosmetic Composition Containing Siloxane-Based Polyamides as Thickening Agents. U.S. Patent.

[16] McDevitt M.R., Chattopadhyay D., Jaggi J.S., Finn R.D., Zanzonico P.B., Villa C., Rey D., Mendenhall J., Batt C.A., Njardarson J.T. (2007). PET Imaging of Soluble Yttrium-86-Labeled Carbon Nanotubes in Mice. PLoS ONE.

[17] Hosoda S., Uemura A. (1992). Effect of the Structural Distribution on the Mechanical Properties of Linear Low-Density Polyethylenes. Polym. J..

[18] Le K. (2018). Textile recycling technologies, colouring and finishing methods. Solid Waste Serv. Vancoover.

[19] Ajith N., Arumugam S., Parthasarathy S., Manupoori S., Janakiraman S. (2020). Global distribution of microplastics and its impact on marine environment—A review. Environ. Sci. Pollut. Res..

[20] O’Brine T., Thompson R.C. (2010). Degradation of plastic carrier bags in the marine environment. Mar. Pollut. Bull..

[21] McNeeley A., Liu Y.A. (2024). Assessment of Nylon-6 Depolymerization for Circular Economy: Kinetic Modeling, Purification, Sustainable Process Design, and Industrial Practice. Ind. Eng. Chem. Res..

[22] Chen X.-H., Wu G., Chen S.-C., Wang Y.-Z. (2023). Facile, high-efficiency, and low-cost depolymerization of PA6 to ϵ-caprolactam enables closed-loop chemical recycling. Polymer.

[23] Ndagano U.N., Cahill L., Smullen C., Gaughran J., Kelleher S.M. (2025). The Current State-of-the-Art of the Processes Involved in the Chemical Recycling of Textile Waste. Molecules.

[24] Andrady A.L. (2015). Chapter 3. Persistence of plastic litter in the oceans. Marine Anthropogenic Litter.

[25] Browne M.A., Crump P., Niven S.J., Teuten E., Tonkin A., Galloway T., Thompson R. (2011). Accumulation of microplastic on shorelines woldwide: Sources and sinks. Environ. Sci. Technol..

[26] Damayanti D., Saputri D.R., Marpaung D.S.S., Yusupandi F., Sanjaya A., Simbolon Y.M., Asmarani W., Ulfa M., Wu H.-S. (2022). Current Prospects for Plastic Waste Treatment. Polymers.

[27] Damayanti, Wu H.S. (2021). Strategic Possibility Routes of Recycled PET. Polymer.

[28] CorbinAlan T.F., Handermann C., Kotek R., Porter W.D., Dellinger J.A., Davis E.A. (1999). Reclaiming Epsilon-Caprolactam from Nylon 6 Carpet. U.S. Patent.

[29] Mihut C., Captain D.K., Gadala-Maria F., Amiridis M.D. (2004). Review: Recycling of nylon from carpet waste. Polym. Eng. Sci..

[30] Eimontas J., Yousef S., Striūgas N., Abdelnaby M.A. (2021). Catalytic pyrolysis kinetic behaviour and TG-FTIR-GC–MS analysis of waste fishing nets over ZSM-5 zeolite catalyst for caprolactam recovery. Renew. Energy.

[31] Skvorčinskienė R., Striūgas N., Navakas R., Paulauskas R., Zakarauskas K., Vorotinskienė L. (2019). Thermal Analysis of Waste Fishing Nets for Polymer Recovery. Waste Biomass Valorization.

[32] Ackermann Y.S., Li W.-J., Op de Hipt L., Niehoff P.-J., Casey W., Polen T., Köbbing S., Ballerstedt H., Wynands B., O’Connor K. (2021). Engineering adipic acid metabolism in Pseudomonas putida. Metab. Eng..

[33] Ragaert K., Delva L., Van Geem K. (2017). Mechanical and chemical recycling of solid plastic waste. Waste Manag..

[34] Hu K., Tian W., Yang Y., Nie G., Zhou P., Wang Y., Duan X., Wang S. (2021). Microplastics remediation in aqueous systems: Strategies and technologies. Water Res..

[35] Zheng L., Wang M., Li Y., Xiong Y., Wu C. (2024). Recycling and Degradation of Polyamides. Molecules.

[36] Zhao Y.-B., Lv X.-D., Ni H.-G. (2018). Solvent-based separation and recycling of waste plastics: A review. Chemosphere.

[37] Fan S.-P., Zakaria S., Chia C.-H., Jamaluddin F., Nabihah S., Liew T.-K., Pua F.-L. (2011). Comparative studies of products obtained from solvolysis liquefaction of oil palm empty fruit bunch fibres using different solvents. Bioresour. Technol..

[38] Iwaya T., Sasaki M., Goto M. (2006). Kinetic analysis for hydrothermal depolymerization of nylon 6. Polym. Degrad. Stab..

[39] Silge J., Robinson D. (2016). tidytext: Text mining and analysis using tidy data principles in R. J. Open Source Softw..

[40] Ye L., Liu X., Beckett K.B., Rothbaum J.O., Lincoln C., Broadbelt L.J., Kratish Y., Marks T.J. (2024). Catalyst metal-ligand design for rapid, selective, and solventless depolymerization of Nylon-6 plastics. Chem.

[41] Al-Salem S.M., Al-Salem S.M. (2019). 3—Energy Production From Plastic Solid Waste (PSW). Plastics to Energy.

[42] Chen H., Wan K., Zhang Y., Wang Y. (2021). Waste to Wealth: Chemical Recycling and Chemical Upcycling of Waste Plastics for a Great Future. ChemSusChem.

[43] Chen J., Liu G., Jin L., Ni P., Li Z., He H., Xu Y., Zhang J., Dong J. (2010). Catalytic hydrolysis of waste nylon 6 to produce ɛ-caprolactam in sub-critical water. J. Anal. Appl. Pyrolysis.

[44] Kumar A., von Wolff N., Rauch M., Zou Y.-Q., Shmul G., Ben-David Y., Leitus G., Avram L., Milstein D. (2020). Hydrogenative Depolymerization of Nylons. J. Am. Chem. Soc..

[45] Kim S., Lee N., Lee J. (2020). Pyrolysis for Nylon 6 Monomer Recovery from Teabag Waste. Polymers.

[46] Kamimura A., Yamamoto S. (2007). An Efficient Method To Depolymerize Polyamide Plastics:  A New Use of Ionic Liquids. Org. Lett..

[47] Yuan X.-X., Zhou Q., Li X.-Y., Yang P., Yang K.-K., Wang Y.-Z. (2014). Degradation of nylon 6 to produce a “pseudo” amino acid ionic liquid. Polym. Degrad. Stab..

[48] Shukla S.R., Harad A.M., Mahato D. (2006). Depolymerization of nylon 6 waste fibers. J. Appl. Polym. Sci..

[49] Wang W., Meng L., Leng K., Huang Y. (2017). Hydrolysis of waste monomer casting nylon catalyzed by solid acids. Polym. Degrad. Stab..

[50] Khuntia S.P., Gadgeel A., Mestry S., Mhaske S.T. (2022). Organo-sulfonic acid catalyzed degradation kinetics and thermodynamic studies of nylon-6 by hydrothermal method. Polym. Adv. Technol..

[51] Wu Y.-H., Wu M.-L., Lin C.-C., Chu W.-L., Yang C.-C., Lin R.T., Deng J.-F. (2012). Determination of caprolactam and 6-aminocaproic acid in human urine using hydrophilic interaction liquid chromatography-tandem mass spectrometry. J. Chromatogr. B.

[52] Fuchs H., Neubauer G., Ritz J., Priester C.-U. (1994). Recovery of Caprolactam from Polycaprolactam. U.S. Patent.

[53] Kembłowski Z., Torzecki J. (1983). Determination of the weight-average molecular weight of polyamide-6 on the basis of melt viscosity. Rheol. Acta.

[54] Wang R., Liu X., Meng C., Wu Y., Zeng C., Zhang S., Ji P., Wang C., Wang H. (2025). Highly efficient de-volatilization of PA6 melt before spinning, enabling oligomer content control and direct melt spinning. Polymer.

[55] Mark J.E. (1999). Polymer Data Handbook.

[56] (2019). Standard Test Methods for Total, Primary, Secondary, and Tertiary Amine Values of Fatty Amines by Alternative Indicator Method. https://www.astm.org/d2074-07r19.html.

[57] Kulkarni R.S., Kanekar P.P. (1998). Bioremediation of ε-Caprolactam from Nylon-6 Waste Water by Use of Pseudomonas aeruginosa MCM B-407. Curr. Microbiol..

[58] Žagar E., Češarek U., Drinčić A., Sitar S., Shlyapnikov I.M., Pahovnik D. (2020). Quantitative Determination of PA6 and/or PA66 Content in Polyamide-Containing Wastes. ACS Sustain. Chem. Eng..

[59] Aquafil S.p.A. (2022). ECONYL^®^ Regeneration System: Closing the Loop for Nylon 6. Sustain. Rep..

[60] DOMO Chemicals GmbH (2024). Chemically Recycled PA6. Sustain. Rep..

[61] BASF SE (2021). From Plastic Waste to Virgin-Grade Products. ChemCycling^TM^ Proj..

[62] Toray Industries, Inc., Honda Motor Co., Ltd. Honda and Toray Begin Joint Demonstration of Closed-loop Recycling of Nylon Resin. News Release, 19 September 2023. https://www.toray.com.

[63] RadiciGroup S.p.A RENYCLE^®^ Let’s Write a New Sustainable Story. https://www.radicigroup.com/en/products/plastics/sustainable-engineering-polymers-renycle.

[64] Williams P.T., Slaney E. (2007). Analysis of products from the pyrolysis and liquefaction of single plastics and waste plastic mixtures. Resour. Conserv. Recycl..

[65] Quartey E.T., Tosefa H., Danquah K.A.B., Obrsalova I. (2015). Theoretical Framework for Plastic Waste Management in Ghana through Extended Producer Responsibility: Case of Sachet Water Waste. Int. J. Environ. Res. Public Health.

[66] Badia J., Ribes-Greus A. (2016). Mechanical recycling of polylactide, upgrading trends and combination of valorization techniques. Eur. Polym. J..

[67] Morales J., Rodrigue D. (2024). The Effect of Reprocessing and Moisture on Polyamide Recycling: A Focus on Neat, Composites, and Blends. Macromol. Mater. Eng..

[68] Liu X., Bertilsson H. (1999). Recycling of ABS and ABS/PC blends. J. Appl. Polym. Sci..

[69] Abdelwahab M.A., Chang B.P., Mohanty A.K., Misra M. (2022). Waste valorization in sustainable engineering materials: Reactive processing of recycled carpets waste with polyamide 6. Polym. Test..

[70] Ellis L.D., Rorrer N.A., Sullivan K.P., Otto M., McGeehan J.E., Román-Leshkov Y., Wierckx N., Beckham G.T. (2021). Chemical and biological catalysis for plastics recycling and upcycling. Nat. Catal..

[71] Georgiopoulou I., Pappa G.D., Vouyiouka S.N., Magoulas K. (2021). Recycling of post-consumer multilayer Tetra Pak^®^ packaging with the Selective Dissolution-Precipitation process. Resour. Conserv. Recycl..

[72] Wan Z., Huang Y., Xiang L., Zhao J., Song Z., Feng H., Zhang S., Wu C. (2025). Molecular Insights into Hydrolysis, Alcoholysis, Ammonolysis, and Acidolysis of Polyamide 6. J. Phys. Chem. A.

[73] Jagodzińska K., Yang W., Jönsson P.G., Forsgren C. (2021). Can torrefaction be a suitable method of enhancing shredder fines recycling?. Waste Manag..

[74] Bockhorn H., Donner S., Gernsbeck M., Hornung A., Hornung U. (2001). Pyrolysis of polyamide 6 under catalytic conditions and its application to reutilization of carpets. J. Anal. Appl. Pyrolysis.

[75] Nikje M.M.A., Nikrah M. (2007). Chemical Recycling and Liquefaction of Rigid Polyurethane Foam Wastes through Microwave Assisted Glycolysis Process. J. Macromol. Sci. Part A.

[76] Mukherjee A., Goel D. (1978). Depolymerization of poly-ϵ-caprolactam catalyzed by sodium hydroxide. J. Appl. Polym. Sci..

[77] Czernik S., Elam C.C., Evans R.J., Meglen R.R., Moens L., Tatsumoto K. (1998). Catalytic pyrolysis of nylon-6 to recover caprolactam. J. Anal. Appl. Pyrolysis.

[78] Bryson L.G. (2008). Monomer Recovery from Nylon Carpets via Reactive Extrusion. Ph.D. Thesis.

[79] Yang W., Jung S., Lee J., Lee S.W., Kim Y.T., Kwon E.E. (2023). Selective recovery of caprolactam from the thermo-catalytic conversion of textile waste over γ-Al_2_O_3_ supported metal catalysts. Environ. Pollut..

[80] Pashaei S., Avval M.M., Syed A.A. (2011). Thermal degradation kinetics of nylon6/GF/crysnano nanoclay nanocomposites by TGA. Chem. Ind. Chem. Eng. Q. CICEQ.

[81] Khedri S., Elyasi S. (2016). Kinetic analysis for thermal cracking of HDPE: A new isoconversional approach. Polym. Degrad. Stab..

[82] Herrera M., Matuschek G., Kettrup A. (2001). Main products and kinetics of the thermal degradation of polyamides. Chemosphere.

[83] Hu S., Jess A., Xu M. (2007). Kinetic study of Chinese biomass slow pyrolysis: Comparison of different kinetic models. Fuel.

[84] Slopiecka K., Bartocci P., Fantozzi F. (2012). Thermogravimetric analysis and kinetic study of poplar wood pyrolysis. Appl. Energy.

[85] Lim A.C.R., Chin B.L.F., Jawad Z.A., Hii K.L. (2016). Kinetic analysis of rice husk pyrolysis using Kissinger-Akahira-Sunose (KAS) method. Procedia Eng..

[86] Eimontas J., Striūgas N., Abdelnaby M.A., Yousef S. (2021). Catalytic Pyrolysis Kinetic Behavior and TG-FTIR-GC–MS Analysis of Metallized Food Packaging Plastics with Different Concentrations of ZSM-5 Zeolite Catalyst. Polymers.

[87] Pannase A.M., Singh R.K., Ruj B., Gupta P. (2020). Decomposition of polyamide via slow pyrolysis: Effect of heating rate and operating temperature on product yield and composition. J. Anal. Appl. Pyrolysis.

[88] Huczkowski P., Kapko J., Olesiak R. (1978). Degradation of nylon-6 in ethylene glycol. Polymer.

[89] Hommez B., Goethals E.J. (1998). Degradation of Nylon-6 By Glycolysis. Part 1: Identification of Degradation Products. J. Macromol. Sci. Part A.

[90] Huczkowski P., Kapko J. (1980). Degradation of nylon-6 in ethylene glycol: 2. Mathematical illustration of degradation. Polymer.

[91] Lan J., Deng C., Zhao Z.-Y., Wang Y.-Z. (2025). An upcycling strategy for polyamide 6: Preparing thermoplastic polyamide elastomers from glycolysates produced by controlled degradation. Green Chem..

[92] Kumar P., Go J., Letteri R., Saito T., Davis R.J. (2026). Novel insight into the kinetics of amide bond glycolysis for Nylon-6 depolymerization. Chem. Eng. J..

[93] Telli A., Özdil N. (2015). Effect of Recycled PET Fibers on the Performance Properties of Knitted Fabrics. J. Eng. Fibers Fabr..

[94] Brodrero S. Chemical recycling of polyamide 6, 6 and polyamide 6 through a two step AMI-/ammonolysis process. Proceedings of the 4th Annual Conference on Recycling of Fibrous Textile and Carpet Waste.

[95] Wang Y., Zhang Y., Polk M., Kumar S., Muzzy J. (2003). Recycling of carpet and textile fibers. Plast. Environ..

[96] Coeck R., De Bruyne A., Borremans T., Stuyck W., De Vos D.E. (2022). Ammonolytic Hydrogenation of Secondary Amides: An Efficient Method for the Recycling of Long-Chain Polyamides. ACS Sustain. Chem. Eng..

[97] Kalfas G.A. (1998). Mathematical Modeling of the Depolymerization of Polyamide Mixtures—Part I: Kinetic Mechanism and Parametric Studies in Batch Reactors. Polym. React. Eng..

[98] Brette M.M., Holm A.H., Drozdov A.D., Christiansen J.d.C. (2024). Pure Hydrolysis of Polyamides: A Comparative Study. Chemistry.

[99] Hernández A.R., Contreras O.C., Acevedo J.C., Navarro Moreno L.G. (2013). Poly(ε-caprolactone) Degradation Under Acidic and Alkaline Conditions. Am. J. Polym. Sci..

[100] Braun M., Levy A., Sifniades S. (1999). Recycling nylon 6 carpet to caprolactam. Polym.-Plast. Technol. Eng..

[101] Jenczewski T.J., Crescentini L., Mayer R.E. (1997). Monomer Recovery from Multi-Component Materials. U.S. Patent.

[102] Moran E.F., McKinney R.J. (1995). Conversion of Nylon 6 and/or Nylon 6, 6 to Adipic Acid. U.S. Patent.

[103] Gama N., Araújo J., Godinho B., Ferreira A., Barros-Timmons A. (2024). Solvolysis of Nylon: A Pathway to Sustainable Recycling and Circular Economy. Sustainability.

[104] Zhang H., Zhao Y., Wang Y., Li R., Tang M., Zeng W., Wang Y., Chang X., Han B., Liu Z. (2024). Valorization of polycaprolactone for the production of nylon-6 monomers. Green Chem..

[105] Pope S., Wæraas A. (2016). CSR-washing is rare: A conceptual framework, literature review, and critique. J. Bus. Ethics.

[106] Khawam A., Flanagan D.R. (2006). Basics and applications of solid-state kinetics: A pharmaceutical perspective. J. Pharm. Sci..

[107] Price D.M., Hourston D.J., Dumont F., Meyers R.A. (2000). Thermogravimetry of polymers. Encyclopedia of Analytical Chemistry.

[108] Beyler C.L., Hirschler M.M. (2002). Thermal decomposition of polymers. SFPE Handbook of Fire Protection Engineering.

[109] Klun U., Kržan A. (2000). Rapid microwave induced depolymerization of polyamide-6. Polymer.

[110] Ludlow-Palafox C., Chase H.A. (2001). Microwave-Induced Pyrolysis of Plastic Wastes. Ind. Eng. Chem. Res..

[111] Chen H., Yang R., Dong B., Sun H., Xu G., Wang Q. (2024). Closed-loop recycling of Nylon-6 to Caprolactam catalyzed by a green and effective phosphazene base. J. Polym. Sci..

[112] Dellinger J.A. (1993). Constant Composition Recycle of Nylon 6 Polymerization Wash Water. U.S. Patent.

[113] Damayanti D., Marpaung D.S.S., Kodarif A.R., Sanjaya A., Saputri D.R., Fahni Y., Rahmiyati L., Silvia P.Z., A’Yuni D.Q., Imalia C.L. (2025). Biocatalytic Recycling of Polyethylene Terephthalate: From Conventional to Innovative Routes for Transforming Plastic and Textile Waste into Renewable Resources. Resources.

[114] Friedrich J., Zalar P., Mohorčič M., Klun U., Kržan A. (2007). Ability of fungi to degrade synthetic polymer nylon-6. Chemosphere.

[115] Bell E.L., Rosetto G., Ingraham M.A., Ramirez K.J., Lincoln C., Clarke R.W., Gado J.E., Lilly J.L., Kucharzyk K.H., Erickson E. (2024). Natural diversity screening, assay development, and characterization of nylon-6 enzymatic depolymerization. Nat. Commun..

[116] Turk S.C., Kloosterman W.P., Ninaber D.K., Kolen K.P., Knutova J., Suir E., Schürmann M., Raemakers-Franken P.C., Müller M., de Wildeman S.M. (2016). Metabolic Engineering toward Sustainable Production of Nylon-6. ACS Synth. Biol..

[117] Meng C., Wu Y., Wang R., Zhang S., Ji P., Wang C., Wang H. (2025). Efficient hydrolytic recycling of PA6 supported by kinetic modeling. Polym. Degrad. Stab..

[118] Zhang Y., Zhan L., Xu Z. (2024). Closed-Loop Upcycling of Waste Nylon Plastic under Hydrothermal Clean Water Atmosphere. Environ. Sci. Technol..

[119] dos Passos J.S., Skibsted S.K.G., Biller P. (2025). Enhancing Recycling of Polyamide 6 and Polyethylene Multilayer Plastics through Sequential Hydrothermal Liquefaction. Energy Fuels.

[120] Cheung E., Alberti C., Bycinskij S., Enthaler S. (2021). Zinc-Catalyzed Chemical Recycling of Poly(ϵ-caprolactone) Applying Transesterification Reactions. ChemistrySelect.

[121] Alberti C., Figueira R., Hofmann M., Koschke S., Enthaler S. (2019). Chemical Recycling of End-of-Life Polyamide 6 via Ring Closing Depolymerization. ChemistrySelect.

[122] Kamimura A., Shiramatsu Y., Kawamoto T. (2019). Depolymerization of polyamide 6 in hydrophilic ionic liquids. Green Energy Environ..

[123] Wursthorn L., Beckett K., Rothbaum J.O., Cywar R.M., Lincoln C., Kratish Y., Marks T.J. (2023). Selective Lanthanide-Organic Catalyzed Depolymerization of Nylon-6 to ϵ-Caprolactam. Angew. Chem..

[124] Fieser M.E., Knight K.D. (2024). Catalyst design strategies for solventless chemical recycling of Nylon-6. Chem.

[125] Bertolla M., Cecchetto M., Dal Moro A., Modesti M., Guerra S. (2024). Process for Producing Epsilon-Caprolactam by Depolymerization of Polycaprolactam (pa6). U.S. Patent.

[126] Češarek U., Pahovnik D., Žagar E. (2020). Chemical Recycling of Aliphatic Polyamides by Microwave-Assisted Hydrolysis for Efficient Monomer Recovery. ACS Sustain. Chem. Eng..

[127] Minor A.-J., Goldhahn R., Rihko-Struckmann L., Sundmacher K., Kokossis A.C., Georgiadis M.C., Pistikopoulos E. (2023). Techno-Economic Process Analysis of the Chemical Recycling of Nylon 6 Using Phosphoric Acid. Computer Aided Chemical Engineering.

[128] Tonsi G., Caccia M., Maesani C., Simon Ostan P., Ortenzi M.A., Alini S., Pirola C. (2025). Polyamide recycling by selective dissolution approach: Life Cycle Assessment study and environmental impacts comparison with different recycling technologies. Polym. Eng. Sci..

[129] Minor A.-J., Goldhahn R., Ganzer C., Lejeune M., Rihko-Struckmann L., Sundmacher K. (2026). Parametric Life Cycle Assessment of Chemical Recycling of Nylon-6 to Caprolactam. Environ. Sci. Technol..

[130] Corbin T.F., Dellinger J.A., Wagener K.B. (1988). Method for Removing Impurities from Caprolactam. U.S. Patent.

[131] Crescentini L., DeCaprio J.D., Fisher W.B., Lilley R.J. (1988). Removal of Light Impurities from Caprolactam by Distillation with Water. U.S. Patent.

[132] Shu Y., Ye L., Yang T. (2008). Study on the long-term thermal-oxidative aging behavior of polyamide 6. J. Appl. Polym. Sci..

[133] Allen N.S., Edge M. (1992). Fundamentals of Polymer Degradation and Stabilization.

[134] Laoutid F., Bonnaud L., Alexandre M., Lopez-Cuesta J.M., Dubois P. (2009). New prospects in flame retardant polymer materials: From fundamentals to nanocomposites. Mater. Sci. Eng. R Rep..

[135] Al-Salem S.M., Lettieri P., Baeyens J. (2009). Recycling and recovery routes of plastic solid waste (PSW): A review. Waste Manag..

[136] Camino G., Costa L. (1988). Performance and mechanisms of fire retardants in polymers—A review. Polym. Degrad. Stab..

[137] Rahman M., Brazel C.S. (2004). The plasticizer market: An assessment of traditional plasticizers and research trends to meet new challenges. Prog. Polym. Sci..

[138] Hunger K. (2007). Industrial Dyes: Chemistry, Properties, Applications.

[139] Pickering S.J. (2006). Recycling technologies for thermoset composite materials—Current status. Compos. Part A Appl. Sci. Manuf..

